# Polymorphisms of Antigen-Presenting Machinery Genes in Non-Small Cell Lung Cancer: Different Impact on Disease Risk and Clinical Parameters in Smokers and Never-Smokers

**DOI:** 10.3389/fimmu.2021.664474

**Published:** 2021-05-31

**Authors:** Andrzej Wiśniewski, Maciej Sobczyński, Konrad Pawełczyk, Irena Porębska, Monika Jasek, Marta Wagner, Wanda Niepiekło-Miniewska, Aneta Kowal, Joanna Dubis, Natalia Jędruchniewicz, Piotr Kuśnierczyk

**Affiliations:** ^1^ Laboratory of Immunogenetics and Tissue Immunology, Hirszfeld Institute of Immunology and Experimental Therapy, Polish Academy of Sciences, Wrocław, Poland; ^2^ Department of Bioinformatics and Genomics, Faculty of Biotechnology, University of Wrocław, Wrocław, Poland; ^3^ Department and Clinic of Thoracic Surgery, Wrocław Medical University, Wrocław, Poland; ^4^ Department of Pulmonology and Lung Oncology, Wrocław Medical University, Wrocław, Poland; ^5^ Research and Development Centre, Regional Specialist Hospital in Wrocław, Wrocław, Poland

**Keywords:** non-small cell lung cancer, smokers *versus* never-smokers, antigen-processing machinery, genetics, *ERAP1*, *ERAP2*, *PSMB9 (LMP2)*

## Abstract

Lung cancer is strongly associated with cigarette smoking; nevertheless some never-smokers develop cancer. Immune eradication of cancer cells is dependent on polymorphisms of HLA class I molecules and antigen-processing machinery (APM) components. We have already published highly significant associations of single nucleotide polymorphisms (SNPs) of the *ERAP1* gene with non-small cell lung cancer (NSCLC) in Chinese, but not in Polish populations. However, the smoking status of participants was not known in the previous study. Here, we compared the distribution of APM polymorphic variants in larger cohorts of Polish patients with NSCLC and controls, stratified according to their smoking status. We found significant but opposite associations in never-smokers and in smokers of all tested SNPs (*rs26653, rs2287987, rs30187*, and *rs27044*) but one (*rs26618*) in *ERAP1*. No significant associations were seen in other genes. Haplotype analysis indicated that the distribution of many *ERAP1/2* haplotypes is opposite, depending on smoking status. Additionally, haplotypic combination of low activity ERAP1 and the lack of an active form of ERAP2 seems to favor the disease in never-smokers. We also revealed interesting associations of some APM polymorphisms with: age at diagnosis (*ERAP1 rs26653*), disease stage (*ERAP1 rs27044*, *PSMB9 rs17587*), overall survival (*ERAP1 rs30187*), and response to chemotherapy (*ERAP1 rs27044*). The results presented here may suggest the important role for ERAP1 in the anti-cancer response, which is different in smokers *versus* never-smokers, depending to some extent on the presence of ERAP2, and affecting NSCLC clinical course.

## Introduction

Lung cancer is the most common cause of cancer death. Among risk factors of lung cancer, smoking is considered to be the predominant risk factor (1, 2). The ratio of mortality to incidence is 0.87, so most patients eventually die of the disease (3). In addition, survival is in inverse proportion to smoking, *i.e.*, decreases with the growing number of pack–years (1). However, epidemiological data indicate that other risk factors exist. First, only about 15% of smokers get lung cancer (2). Second, this disease appears also, albeit much less frequently (10–25% of all lung cancer cases) among never-smokers (4–6). Although lung cancer develops in never-smokers worldwide, geographic variation is striking, with 30 to 40% of Asian patients with lung cancer being never-smokers, compared with 10 to 20% of Caucasian patients (2). Non-small cell lung cancer (NSCLC) type accounts for approximately 85% of all cases of lung cancer, especially in never-smokers (almost all cases), and adenocarcinoma is a major type of NSCLC (7–9). However, accurate data on the incidence of lung cancer in never-smokers are rare due to the lack of information about smoking habits in the majority of cancer registries (10).

Malignant cells are eliminated by both innate and adaptive immunity, which are dependent on the HLA class I (HLA-I) molecule expression level on the surface of tumor cells (11). Innate natural killer cells, with their KIR or NKG2/CD94 receptors, recognize the markedly reduced amount or lack of HLA class I molecules which frequently happens on tumor cells (12). The efficient adaptive immunity against tumors is mediated by CD8+ cytotoxic T lymphocytes (CTLs) which recognize tumor neoantigens presented to their specific T cell receptors by HLA-I molecules (13). Antigen presentation by HLA-I glycoproteins depends (i) on genetics of the extremely polymorphic HLA-I molecules themselves, (ii) on differential expression and/or activity of antigen-processing machinery (APM) elements due to some polymorphisms of their genes, and (iii) on regulatory effects of other molecules. Associations of HLA and other genes with human diseases, including neoplasms, have already been studied for decades (14).

Peptides bound and presented by HLA-I molecules are prepared by the antigen processing machinery (APM) composed of multiple components. Proteins synthesized in the cytosol, but not transported to the site of their destiny, are polyubiquitinated and degraded in the proteasome—a multiprotein proteolytic complex in the cytosol. Under conditions of immune response, some protein subunits of the proteasome are replaced by functionally different counterparts—PSMB9, PSMB8, and PSMB10 (called PSMB for “proteasome 20S subunit beta” or, according to older nomenclature, LMP2, LMP7, and LMP10 for “low-molecular-mass polypeptide”) and such a protein complex is called “immunoproteasome”. Both proteasome and immunoproteasome proteolytically cut protein chains into peptides which are transported to the endoplasmic reticulum by the transporter associated with antigen processing (TAP) molecules (consisting of TAP1 and TAP2 subunits) (13). However, peptides produced by immunoproteasome fit TAP-mediated transport and MHC-I binding better than peptides produced by a common proteasome (13, 15). Then, ERAP1 (endoplasmic reticulum aminopeptidase associated with antigen presentation 1) and ERAP2 trim the transported peptides (if these are too long) to a suitable length (usually 8–10 amino acids). Empty HLA class I molecules bind the peptides with the help of other molecules present in the endoplasmic reticulum. Finally, HLA-I/peptide complexes are transported to the cell surface, where they may be recognized by CD8+ T cells *via* their specific T cell receptors (13–15) or by NK cells *via* KIRs and CD94/NKG2 (16, 17).

Defects in APM may enable tumor cells to evade T lymphocyte-mediated recognition and lysis, which has already been shown for multiple tumor types (13). Disturbances of APM component expression have been shown for several types of human tumors (18). For example, PSMB, TAP, and/or ERAP molecules were down- or up-regulated in different cancers (19–27). Similarly, loss, retention or acquisition as well as imbalances of ERAP1 and ERAP2 expression in different solid tumor tissues as compared to normal counterparts has been observed (28) which may affect disease outcome and response to treatment (29).

Importantly, genes encoding APM molecules exhibit some levels of polymorphism, and some genetic variations may result in changes in expression or activity of these molecules (14). Moreover, some variants were found to be associated with human diseases, including neoplasms (30). For example, *PSMB8* variant encoding a protein with lysine in position 49 (PSMB8-K) has a lower mRNA stability and a lower interferon-gamma-mediated induction than an alternative form with glutamine in this position (PSMB8-Q) and was associated with colon cancer (31). Although polymorphism of *TAP1* and *TAP2* genes is relatively low, some variants have been associated with cancer risk. For instance, the *rs1057141*/TAP1-I393V variation was positively associated with multiple myeloma (32) and higher risk of cervical intraepithelial neoplasia after HPV infection (33). Furthermore, two *TAP1* polymorphisms: *rs1057141* and *rs1135216* [D697G] were associated with high-grade cervical intraepithelial carcinoma (34). With regard to *TAP2*, heterozygous genotype GA of the SNP *rs2228396* [A565T] was associated with susceptibility to chronic lymphoid leukemia (CLL), whereas *rs241447* G allele [T665A] was a risk factor for chronic myeloid leukemia and multiple myeloma alongside CLL (32). *ERAP* genes, particularly *ERAP1*, are polymorphic, which influences their expression, activity and substrate specificity (35, 36) as well as the HLA class I-bound immunopeptidome repertoire (37). According to this, several *ERAP1* polymorphisms were described as affecting susceptibility to cervical carcinoma in Dutch (38) and Indonesian (39) populations.

Polymorphisms of antigen-processing machinery genes were not tested so far in non-small cell lung cancer, except for our previous report on the *ERAP1* gene showing highly significant associations in Chinese but not in Polish populations (40). However, smoking status was not established in these two cohorts. Therefore, we hypothesized that genetic polymorphisms of the APM components (*PSMB8, PSMB9, TAP1, TAP2, ERAP1, ERAP2*) might be associated with NSCLC also in Poles, but rather in never-smokers, devoid of this strong environmental factor. Results presented here show that for *ERAP1*, four out of the five examined SNPs were significantly associated with NSCLC, not only in never-smokers but surprisingly also in smokers, however in opposite directions. In addition, three studied polymorphisms in *ERAP1* and one in *PSMB9* were also associated with some clinical characteristics.

## Materials and Methods

### Study Subjects

A total of 464 newly diagnosed patients with pathologically documented NSCLC (according to WHO criteria) were enrolled in our study by the Department of Pulmonology and Lung Cancer, Wrocław Medical University, Wrocław and by the Thoracic Surgery Center, Lower Silesian Centre of Lung Diseases, Wroclaw. The histological type of lung cancer was identified according to the World Health Organization (WHO 2015) classifications. Pathologic stages were determined according to the International System for Staging Lung Cancer (41) as described (42). According to histopathological reports, NSCLC included adenocarcinoma (AC), squamous cell carcinoma (SCC), and a few cases of large cell carcinoma. In several cases, a detailed subtype of NSCLC was not determined due to small cytological specimen and/or only palliative regimen of treatment. NSCLC patients with a history of primary cancer other than lung cancer were excluded from this study. The study also did not include palliative patients whose stage of the disease was not established due to the general condition and/or coexisting serious unstable diseases. Advancement of the disease was determined according to the TNM staging system (43) based on radiological exam and endoscopic techniques. Chest computed tomography, PET-CT, and as needed brain MRI/CT, and bone scintigraphy were done. All patients underwent bronchofiberoscopy. If it was necessary to verify the status of mediastinal lymph nodes, additional EBUS-TBNA or mediastinoscopy was applied. In the case of curative surgery the definite clinical stage was verified by histopathological examination of postoperative specimens especially those of the lymph nodes. The ECOG scale was used to assess the general condition and quality of life of patients. Patients underwent surgery with adjuvant or neoadjuvant chemotherapy (two/three courses given at three-week intervals) as needed, radiotherapy, radiochemotherapy, chemotherapy, or palliative treatment only, depending on disease stage based on local recommendations. The treatment response was based on radiological examinations including mainly chest computed tomography evaluation (CT, Response Evaluation Criteria in Solid Tumors) or chest X-rays, if chest CTs were not available, with clinical and radiological data monitoring appearance of distant metastases (other lung, pleura, central nervous system, bones, adrenal glands) in advanced stage. Tumor response was assessed every 2 months during the first year after diagnosis, every 3 months between 12 and 18 months, and thereafter the interval of assessment was at the physician’s discretion. Overall survival was assessed from the date of NSCLC diagnosis until death from any cause or until 2 years when data collection was finished. Detailed characteristics of the patients are shown in **Table 1**.

**Table 1:** Clinical characteristics of investigated patients.

	Gender	0^1^	1−10	11−20	21−30	31−40	>40
Smoking history expressed in pack–years	Men (n = 322)	29	2	38	77	*90*	86
	%	*9*	*0.6*	*11.8*	*23.9*	*28*	*26.7*
	% cumulative	*9*	*9.6*	*21.4*	*45.3*	*73.3*	*100*
	Women (n = 142)	31	3	33	*32*	32	11
	%	*21.8*	*2.1*	*23.2*	*22.5*	*22.5*	*7.7*
	% cumulative	*21.8*	*23.9*	*47.1*	*69.6*	*92.1*	*100*
Age at diagnosis	**Gender**	**Min**	**Q1**	**Median**	**Sn**	**Q3**	**Max**
	Men	44	59	64	7	70	93
	Women	35	57	63	8	69	86
Histological type	**Gender**	**Squamous cell carcinoma**		**Adenocarcinoma**		**Large cell carcinoma**	
		n	%	n	%	n	%
	Men	99	*51*	79	*40.7*	16	*8.2*
	Women	31	*33.3*	55	*59.1*	7	*7.5*
Staging	**Gender**	**I**	**II**	**III**	**IV**	**Median**	**Q1**
	Men	65	50	95	108	III	II
	%	*20.4*	*15.7*	*29.9*	*34*		
	% cumulative	20.4	*36.1*	*66*	*100%*		
	Women	45	15	39	40	III	I
	%	*32.4*	*10.8*	*28.1*	*28.8*		
	% cumulative	32.4	*43.2*	*71.3*	*100%*		
ECOG	**Gender**	**0**	**1**	**2**	**3**	**4**	**Median**
	Men	66	95	27	20	5	1
	%	*31*	*44.6*	*12.7*	*9.4*	*2.3*	
	% cumulative	*31*	*75.6*	*88.3*	*97.7*	*100*	
	Women	47	37	9	8	1	1
	%	*46.1*	*36.3*	*8.8*	*7.8*	*1*	
	% cumulative	*46.1*	*82.4*	*91.2*	*99*	*100*	
Therapy	**Surgery**	**Chemotherapy**	**Radiotherapy**	**Only surgery**	**Only chemotherapy**	**Only surgery and chemotherapy**	**Palliative therapy**
n	216	211	26	74	121	52	36
%	*55.4*	*54.1*	*8.4*	*23.9*	*39.1*	*16.8*	*11.7*
Response to therapy	**Complete response**	**Partial response**	**Stable disease**	**Progressive disease**	**∑**	**Any response**	**No progression**
N	53	57	23	60	193	110	133
%	*27.5*	*29.5*	*11.9*	*31.1*	*100%*	*57%*	*68.9%*
Survival (months)	**Gender**	**Min**	**Q1**	**Median**	**Sn**	**Q3**	**Max**
All observed	Men	0.3	5.7	12	9.2	23.1	116
	Women	1	8.7	21.5	11.8	29	139
Not death	Men	3.4	12.9	24.4	9.9	30	116
	Women	3.5	17.9	25.6	9	31	139
Death	Men	0.3	3.25	8	6.2	13	53
	Women	1	4	8	6.6	16.6	24.4

Q1, Q3, first and third quartiles (cut off at 25% and 75%, respectively); Sn, robust measure of variability—typical absolute difference between two randomly chosen persons; ^1^—never-smokers.

A total of 409 unrelated healthy Polish individuals (42 females and 367 males, for details see **Supplementary Table 1**), mostly from the same geographic region (Lower Silesia), were taken as a control group.

Both patients and controls were interviewed for their history of smoking and divided into never-smokers (0 pack–years) and smokers (present or past smokers—quitting at least one year before diagnosis). Additionally for each patient we had information about the number of pack–years. Information about smoking history for patients and controls was presented in **Table 1** and in **Supplementary Table 2**, respectively. As it can be seen from **Table 1**, women with NSCLC generally smoked a lower amount of cigarettes than men (*e.g.* 47.1% of women smoked up to 11–20 pack–years, while among men it was only 21.4%, *i.e* almost 80% of men smoked more than 11–20 p-y), had lower disease stage, had slightly lower values on the ECOG scale, and had lower risk of death than men of the same age, and the same disease stage.

### DNA Isolation and SNP Genotyping

All blood samples were collected before treatment. Genomic DNA was extracted from 2 ml of frozen blood using an QIAamp DNA Blood Midi Kit (Qiagen) following the manufacturer’s instruction. All 17 SNPs in *ERAP1* (*rs26653, rs26618, rs2287987, rs30187, rs27044*), *ERAP2* (*rs2248374, rs2549782*), *TAP1* (*rs1135216, rs1057141*), *TAP2* (*rs4148876, rs1800454, rs241447, rs16870908*), *PSMB9* (*rs1351383, rs2127675, rs17587*, and *PSMB8* (*rs2071543*) were selected based on literature data and genotyped using the TaqMan SNP Genotyping Assays (Applied Biosystems, Foster City, USA) according to the manufacturer’s instruction. Relevant information about each SNP including assay IDs was provided in **Supplementary Table 3**. Roche LightCycler 480 II Real Time PCR System was used to conduct PCR reactions and allelic discrimination.

### Statistical Analysis

Genotype distributions among Cases and Controls and conditional association between SNP genotype and cancer were analyzed after adjusting for smoking as the stratification variable. Strength of association was measured with odds ratios (ORs) for smokers and never-smokers with 95%-confidence interval. In the case of stable association among strata, *i.e. OR_smokers_*≈ *OR_never-smokers_* association was measured with common odds ratio and estimated with Mantel–Heanszel estimator (OR.MH). OR.MH is useful when OR seems stable among smokers and never-smokers. This homogeneity of ORs was tested as hypothesis H0: *OR_smokers_*≈ *OR_never-smokers_ vs*. H1: *OR_smokers_*≠ *OR_never-smokers_* and p-value is reported (*p_MH_*) as the result of Breslow test. If true odds ratios among two strata are not identical but do not vary much, OR.MH still is a useful summary of the conditional associations between SNP and risk of cancer. Departure from the Hardy–Weinberg equilibrium was tested with chi-square test and measured as $f=\frac{p_{cc}-p_{c}^{2}}{p_{c}(1-p_{c})}$ where p_c_ and p_cc_ are allele c and genotype cc frequencies. *f* < 0 in case of deficiency of homozygotes, *f* > 0 corresponds to deficiency of heterozygotes and *f* = 0 when locus is in HWE. Difference in genotype distributions between patients and controls was tested with the chi-square test on one degree of freedom (score test). Based on properties of gamma family distributions, test for null H0: *All four ORs = 1 both in the smokers and in the never-smokers group* opposite to $\text{H}1:\exists \;i\in \{1,\;2,\;3,\;4\}:OR_{i}\neq 1$ was tested with chi-square statistic on *k* = 2 degrees of freedom and reported in **Table 2** as P^*^. Global statistic for association of SNPs of a gene with the risk of cancer has $\chi_{2\times L}^{2}$ distribution, where *L* is the number of analyzed SNP in a gene. This procedure tests $\text{H}0:\forall l\in \{1,2,\ldots ,L\}\wedge \forall i\in \{1,2,3,4\}:OR_{l,i}=1$, opposite to *H*1: ∃*l* ∧ ∃*i*: *OR_l,i_* ≠ 1. Basic statistics used to describe the main variables considered in the paper were median, first and third quartiles (Q1, Q3), minimal and maximal observations. Median was used as a location parameter, and S_n_ statistic was computed as a measure of variability (44) *S_n_* = *med* {*med*|*x_i_* – *x_j_*|; *j* = 1…*n*} which is an average difference between two randomly sampled observations. Expected age of diagnosis depending on genotype in SNPs related with risk of NSCLC was analyzed with F-test of ANOVA for contrasts constructed based on the risks (ORs) of NSCLC and adjusted to sex and smoking status. R^2^ is determination coefficient of the model. Haplotype frequencies were estimated iteratively by maximization of *a posteriori* probabilities. Test for association of NSCLC with haplotypes is chi-squared distributed. Survival was analyzed with proportional hazards models with survival function estimated with Kaplan–Meier estimator. Stage of NSCLC was analyzed with Monte Carlo simulation and tested with Mahalanobis D^2^ statistic. Response to chemotherapy was analyzed with logistic model defined as *h*[*P*(*Y ≤ r*|**x**)] = *α_r_* + **β** *^T^* **x**, where *Y* is type of response, *r* is score for type of response: progressive disease, stable disease, partial response or complete response, x is vector of predictors and *h* is the logit function. In case of small samples confidence intervals CI95% was estimated with *bootstrap* method. **Supplementary Table 4** and **Supplementary Figure 1** present power estimation of the test for association between genotype and risk of cancer.

**Table 2:** Genotypes distributions among NSCLC patients and controls and conditional association between SNP genotype and cancer after adjusting for smoking as the stratification variable.

Gene	SNP	Smoking	n, %	Patients			Controls			OR (CI95%)		OR.MH (CI95%)		P*
				CC	GC	GG	CC	GC	GG	GC/ GG	CC/ GG	GC/ GG	CC/ GG	
*ERAP1*	*rs26653*	smokers	n	24	158	205	4	73	96	**1.01** (0.69; 1.47)	**2.56** (1.07; 10.1)	**0.85** (0.62; 1.18)	**1.21** (0.65; 2.32)	0.011
			%	*6.2*	*40.8*	*53.0*	*2.3*	*42.2*	*55.5*					
		never-smokers	n	3	17	37	16	67	73	**0.51** (0.25; 0.97)	**0.42** (0.06; 1.17)			
			%	*5.3*	*29.8*	*64.9*	*10.3*	*42.9*	*46.8*					
		HWE (p-val, f)		S (p = 0.376; f = −0.05); NS (p = 0.59; f = 0.07)			S (p = 0.019; f = −0.18); NS (p = 0.919; f = 0.01)			*OR_smokers_*≈ *OR_never-smokers,_ p_MH_* = 0.014				
*ERAP 1*	*rs26618*	**Smoking**	**n, %**	**CC**	**TC**	**TT**	**CC**	**TC**	**TT**	**TC/ TT**	**CC/ TT**	**TC/ TT**	**CC/ TT**	0.182
		smokers	n	21	158	210	12	73	86	**0.89** (0.61; 1.29)	**0.71** (0.34; 1.61)	**0.95** (0.69; 1.31)	**1.07** (0.57; 2.15)	
			%	*5.4*	*40.6*	*54.0*	*7.0*	*42.7*	*50.3*					
		never-smokers	n	6	19	32	6	50	100	**1.19** (0.61; 2.29)	**3.09** (0.87; 11)			
			%	*10.5*	*33.3*	*56.1*	*3.8*	*32.1*	*64.1*					
		HWE (p-val, f)		S (p = 0.211; f = −0.06); NS (p=0.236; f=0.16)			S (p = 0.509; f = −0.05); NS (p=0.943; f=-0.01)			*OR_smokers_*≈ *OR_never-smokers,_ p_MH_* = 0.072				
*ERAP 1*	*rs2287987*	**Smoking**	**n, %**	**CC**	**TC**	**TT**	**CC**	**TC**	**TT**	**TC/ TT**	**CC/ TT**	**TC/TT**	**CC/TT**	0.036
		smokers	n	19	121	228	15	56	100	**0.95** (0.64; 1.42)	**0.55** (0.27; 1.15)	**1.14** (0.81; 1.59)	**0.8** (0.41; 1.55)	
			%	*5.2*	*32.9*	*62.0*	*8.8*	*32.7*	*58.5*					
		never-smokers	n	4	25	28	5	49	102	**1.85** (0.98; 3.53)	**2.94** (0.63; 12.31)			
			%	*7.0*	*43.9*	*49.1*	*3.2*	*31.4*	*65.4*					
		HWE (p-val, f)		S (p = 0.568; f = 0.03); NS (p = 0.606; f = −0.07)			S (p = 0.082; f = 0.13); NS (p = 0.759; f = −0.02)			*OR_smokers_*≈ *OR_never-smokers,_ p_MH_* = 0.015				
*ERAP 1*	*rs30187*	**Smoking**	**n, %**	**TT**	**CT**	**CC**	**TT**	**CT**	**CC**	**CT/CC**	**TT/CC**	**CT/CC**	**TT/CC**	0.017
		smokers	n	41	174	175	14	72	85	**1.17** (0.81; 1.72)	**1.39** (0.75; 2.94)	**0.98** (0.71; 1.35)	**0.86** (0.54; 1.42)	
			%	*10.5*	*44.6*	*44.9*	*8.2*	*42.1*	*49.7*					
		never-smokers	n	5	21	31	31	68	57	**0.57 **(0.29; 1.09)	**0.32** (0.09; 0.77)			
			%	*8.8*	*36.8*	*54.4*	*19.9*	*43.6*	*36.5*					
		HWE (p-val, f)		S (p = 0.806; f = −0.01); NS (p = 0.598; f = 0.07)			S (p = 0.824; f = −0.02); NS (p = 0.198; f = 0.1)			*OR_smokers_*≈ *OR_never-smokers,_ p_MH_* = 0.008				
*ERAP 1*	*rs27044*	**Smoking**	**n, %**	**GG**	**CG**	**CC**	**GG**	**CG**	**CC**	**CG/CC**	**GG/CC**	**CG/CC**	**GG/CC**	0.069
		smokers	n	28	147	212	8	61	102	**1.16** (0.79; 1.70)	**1.62** (0.78; 4.29)	**1.06** (0.77; 1.48)	**0.92** (0.53; 1.63)	
			%	*7.2*	*38.0*	*54.8*	*4.7*	*35.7*	*59.6*					
		never-smokers	n	2	21	34	21	57	77	**0.84** (0.43; 1.57)	**0.26** (0.04; 0.73)			
			%	*3.5*	*36.8*	*59.6*	*13.5*	*36.8*	*49.7*					
		HWE (p-val, f)		S (p = 0.713; f = 0.02); NS (p=0.561; f = −0.08)			S (p = 0.771; f = −0.02); NS (p = 0.056; f = 0.15)			*OR_smokers_*≈ *OR_never-smokers,_ P_MH_* = 0.044				
*ERAP 2*	*rs2248374*	**Smoking**	**n, %**	**AA**	**GA**	**GG**	**AA**	**GA**	**GG**	**GA/GG**	**AA/GG**	**GA/GG**	**AA/GG**	0.861
		smokers	n	95	189	100	38	92	41	**0.85** (0.54; 1.31)	**1.02** (0.61; 1.74)	**0.87** (0.59; 1.26)	**1.09** (0.7; 1.7)	
			%	*24.7*	*49.2*	*26.0*	*22.2*	*53.8*	*24.0*					
		never-smokers	n	15	26	16	33	78	45	**0.93** (0.47; 1.96)	**1.28** (0.54; 2.93)			
			%	*26.3*	*45.6*	*28.1*	*21.2*	*50.0*	*28.8*					
		HWE (p-val, f)		S (p = 0.778; f = 0.02); NS (p = 0.507; f = 0.09)			S (p = 0.308; f = −0.08); NS(p=0.937; f = −0.01)			*OR_smokers_*≈ *OR_never-smokers,_ p_MH_* = 0.885				

Strength of association is measured with odds ratios (OR) for smokers (S) and never-smokers (NS) with 95%-confidence interval. OR.MH is common odds ratio estimated with the Mantel and Haenszel estimator which is useful when OR seems stable among smokers and never-smokers. This homogeneity of ORs was tested as H0: OR_smokers_ = OR_never-smokers_ vs. H1: OR_smokers_ ≠ OR_never-smokers_ and p-value is reported (p_MH_). If true odds ratios among two strata are not identical but do not vary much, OR.MH still is a useful summary of the conditional associations between SNP and risk of cancer. The table also presents results of testing hypothesis H0: There are no associations between genotype and risk of cancer, i.e. all OR_S_ = 1 opposite alternative H1: H0 is false, and reports them as P*-values.

## Results

### Genetic Data

**Table 2** shows the analysis of associations between the prevalence of NSCLC and tested SNPs.

#### *ERAP1 rs26653, rs26618, rs2287987, rs30187*, and *rs27044*

We observed different associations of *rs26653*G>C with NSCLC depending on smoking status. In the group of smokers, GC heterozygotes had the same risk as individuals with GG genotype (OR = 1.01; CI95% 0.69; 1.47); however for CC homozygotes a 2.5-fold higher risk of cancer development as compared to the reference group (GG), (OR = 2.56; CI95% 1.07; 10.1), was observed. In never-smokers, on the contrary, the presence of one C allele (GC genotype) was associated with two-fold lower risk as compared to GG homozygotes. The presence of the second C allele was associated with an even lower (1/0.42 ≈ 2.4 times) risk of disease as compared to GG genotype. Additive model for comparison of CC *versus* GG would predict OR ≈ 0.26, and observed OR = 0.42, with confidence intervals CI95% (0.06; 1.17) does not contradict the additive model in this case. Therefore, in never-smokers the presence of one C allele reduced the disease risk by a factor of two. As it was shown in **Table 2**, homogeneity of odds ratios among smokers and never-smokers was tested. In the case of *rs26653* the p-value is reported as *p_MH_* = 0.014. Again it is clear that association between this SNP is not the same in smokers and never-smokers. We also tested the null hypothesis that there is no association between genotype in this SNP and risk of cancer neither in the smokers nor in the never-smokers. Test for this null hypothesis gives the result P^*^ = 0.011. Concluding, there is relationship between genotype in *rs26653*G>C and risk of NSCLC, and this relationship depends on smoking status.

Similarly, in the case of *rs30187* and *rs27044*, we observed that along with the growing number of minor alleles the risk of NSCLC increased in smokers whereas decreased in never-smokers. These results are not surprising because of the existence of linkage disequilibrium between analyzed polymorphisms in *ERAP1* (see **Supplementary Table 7**). In contrast, for *rs2287987* we observed a reverse effect as minor homozygotes protected smokers and predisposed never-smokers (**Table 2**).

Global test for association of all *ERAP1* polymorphisms with lung cancer gives $\chi_{df=10}^{2}=32.6,p=0.000314$ It is worth noting that this association can be discovered in the Caucasian population only after taking into account information about smoking history.

#### ERAP2 rs2248374

The *rs2248374G>A* was distributed similarly in smoking and never-smoking patients and controls (**Table 2**), therefore not showing any significant association with NSCLC by itself, but seemed to influence the effect of ERAP1 (see below). We investigated also another polymorphism of *ERAP2*, namely *rs2549782*. However, because of the nearly complete LD detected between both SNPs in our population (R^2^ = 0.99), we did not include *rs2549782* into the statistical analysis.

#### *ERAP1,2* Haplotype Distribution in Smoking and Never-Smoking Patients and Controls

As genes for ERAP1 and ERAP2 aminopeptidases are located close to each other on chromosome 5, and both enzymes may function not only separately but also in a complex (45, and references therein), which can change their activity and substrate specificity, we analyzed frequencies of *ERAP1* (five SNPs) plus *ERAP2* (one SNP) haplotypes in our groups of patients and controls depending on their smoking status. Details of this analysis are shown in **Tables 3** and **4**. Associations of individual haplotypes with NSCLC were different between smokers and never-smokers. Overall, our analysis suggested that observable effects of haplotypes, like those of individual SNPs, were more distinct in never-smokers. In this group, we detected a significant difference in the frequency of haplotypes between patients and controls ($\chi_{\text{df}=14}^{2}=24.57;\text{p}=0.039$) (**Table 4**). The haplotype *rs26653G-rs26618T-rs2287987C-rs30187C-rs27044C-rs2248374G* increased nearly two-fold (OR = 1.99) the risk of NSCLC, whereas the presence of the haplotype *rs26653C-26618T-rs2287987T-rs30187T-rs27044G-rs2248374G* decreased the disease risk two-fold (OR = 0.52).

**Table 3:** Haplotype frequencies in patients and control group among smokers. Haplotypes were sorted by control frequencies.

Haplotype^1^	Patients Frequencies %	Controls Frequencies %	OR	CI95%	
				2.5%	97.5%
G-C-T-C-C-A	21.6	21.6	1.02	0.73	1.41
G-T-C-C-C-A	11.1	14.4	0.76	0.48	1.17
C-T-T-T-G-G	10.0	9.9	1.05	0.63	1.69
G-T-C-C-C-G	9.9	9.3	1.13	0.67	1.84
G-T-T-T-G-G	12.2	8.4	1.60	0.89	2.85
G-T-T-C-C-G	6.7	7.5	0.93	0.51	1.66
G-C-T-C-C-G	3.4	6.4	0.56	0.25	1.10
C-T-T-C-C-G	5.8	5.3	1.24	0.58	2.62
G-T-T-T-G-A	3.0	4.3	0.81	0.28	1.94
C-T-T-T-C-A	4.5	3.8	1.40	0.56	3.39
G-T-T-C-C-A	4.4	3.7	1.38	0.53	3.21
C-T-T-T-C-G	2.5	3.5	0.85	0.29	2.14
C-T-T-C-C-A	2.6	1.2	1.72	0.64	3.38
Total	97.7%	99.3%	–	–	–
$\chi_{df=13}^{2}=13.995;p=0.3742$					

^1^rs26653-rs26618-rs2287987-rs30187-rs27044-rs2248374.

**Table 4:** Haplotype frequencies in cases and control group among never-smokers. Haplotypes were sorted by control frequencies.

Haplotype^1^	Patients Frequencies %	Controls Frequencies %	OR	CI95%	
				2.5%	97.5%
G-C-T-C-C-A	19.0	17.4	1.21	0.58	2.11
C-T-T-T-G-G	7.2	14	0.52	0.14	1.06
G-T-T-T-G-G	6.3	10.6	0.64	0.14	1.51
G-T-T-C-C-G	8.7	10.5	0.90	0.28	1.90
G-T-C-C-C-A	12.9	9.3	1.62	0.45	3.54
G-T-C-C-C-G	15	9.1	1.99	0.61	4.15
C-T-T-T-C-A	2.2	6.7	0.40	0.13	0.99
G-T-T-T-G-A	5	5.2	1.14	0.22	3.07
C-T-T-T-C-G	3.8	3.6	1.31	0.28	3.78
G-T-T-C-C-A	3.5	3.1	1.42	0.27	4.31
C-T-T-C-C-G	2.7	3.1	1.34	0.27	4.04
C-T-T-C-C-A	3.4	3.1	1.38	0.31	3.91
G-C-T-C-C-G	7.1	2.3	3.71	0.90	9.15
C-T-T-T-G-A	3.2	1.5	2.14	0.48	5.73
Total	100%	99.5%	–	–	–
$\chi_{df=14}^{2}=24.57;p=0.03906$					

^1^rs26653-rs26618-rs2287987-rs30187-rs27044-rs2248374.

In contrast, in smokers we did not find significant differences in haplotype frequencies between patients and controls ($\chi_{\text{df}=13}^{2}=13.995;\text{p}=0.374$) (**Table 3**).

Although none of the haplotypes reached a level of significance in smokers, the effects of some haplotypes were quite different in smokers and never-smokers. The most striking difference was seen for the haplotype *rs26653G-rs26618C-rs2287987T-rs30187C-rs27044C-rs2248374G* which increased the disease risk in never-smokers nearly four-fold (OR = 3.71), but turned out to be protective in smokers (OR = 0.56) (**Tables 3** and **4**). Of note, the other haplotype, *rs26653G-rs26618C-rs2287987T-rs30187C-rs27044C-rs2248374A*, possessing the same alleles in all *ERAP1* SNPs but differing only by *ERAP2* allele, was not associated with NSCLC independently of smoking status.

**Figure 1:**
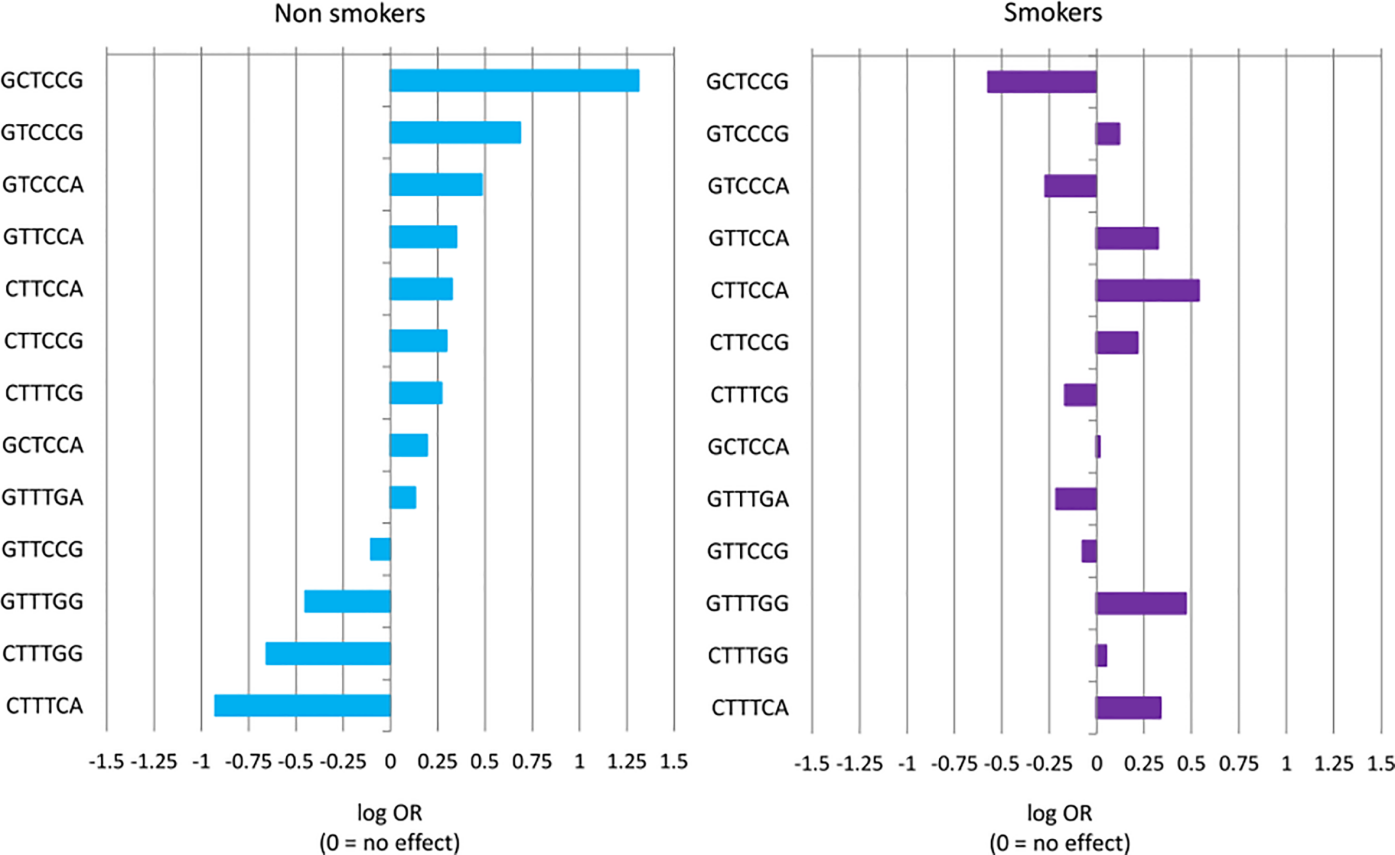
Haplotype effect size of risk of NSCLC measured with log OR and sorted by effect in never-smokers group. A negative value of log OR (log OR < 0) corresponds to a protective effect of haplotype (OR < 1), while log OR > 0 corresponds to a predisposing haplotype effect (OR > 1). Differences in haplotype distributions between cases and controls were tested for smokers (*p* = 0.3742) and never-smokers (*p* = 0.03906).

The effects of individual *ERAP1,2* haplotypes on NSCLC risk in never-smokers and smokers are shown also in **Figure 1**, where the effect size of single haplotypes was measured with log ORs. It clearly shows that majority of haplotypes behaved in an opposing way depending on smoking status.

#### *TAP1 rs1135216* and *rs1057141, TAP2 rs4148876, rs1800454, rs241447* and *rs16870908, PSMB9 rs1351383* and *rs17587, PSMB8 rs2071543*

All these SNPs were distributed similarly in smoking and never-smoking patients and controls (**Supplementary Table 5**).

### Associations of SNPs With Clinical Features

#### Age at Diagnosis and *ERAP1* Polymorphism

**Figure 2:**
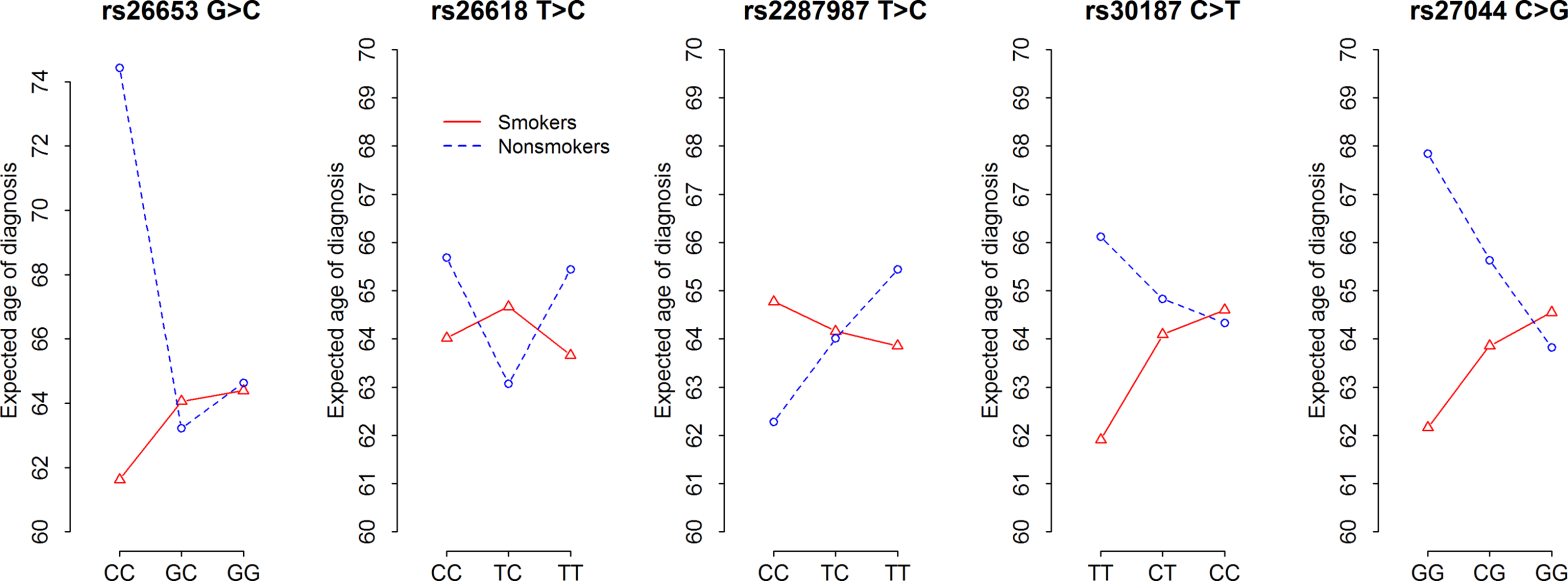
Expected age at diagnosis of NSCLC according to five *ERAP1* SNPs and smoking status. F-statistics and p-values of ANOVA tests for contrasts presented in**Supplementary Table 6**.

Our analysis revealed that *ERAP1* polymorphism influenced age at diagnosis (AAD) of NSCLC depending on smoking status. **Figure 2** and **Supplementary Table 6** show the expected age of diagnosis for each *ERAP1* SNP genotype that was calculated on the basis of observed values, adjusted to gender and smoking. Overall, SNP genotypes associated with disease risk in smokers (see **Table 2**) were also associated with earlier AAD in this group, whereas opposite associations of the same genotypes in never-smokers correlated with later age of diagnosis (**Figure 2**). The strongest relationship was found for *ERAP1 rs26653* (p = 0.0087). In smokers, the disease risk for genotypes GG and GC was nearly the same (OR = 1.0 and 1.01 respectively, **Table 2**), and the expected AAD for these genotypes was also very similar, 64.06 and 64.39 years, respectively (**Supplementary Table 6**). On the other hand, the mean age at diagnosis for the most risky genotype in smokers - *rs26653CC* (OR = 2.56), was lower and achieved the value of 61.62 years. In never-smokers the genotype *rs26653CC* that exerted a strong protective effect (OR = 0.42) elevated the expected age at diagnosis up to the value of 74.44. For comparison, the age at diagnosis for GG and GC genotypes had the values of 64.63 and 63.22, respectively (**Figure 2** and **Supplementary Table 6**).

A similar effect to that observed for *rs26653*, albeit weaker, was demonstrated for *rs30187* and *rs27044*. Thus, *rs30187TT* and *rs27044GG*, bearing some disease risk in smokers, were associated with lower age at diagnosis values, whereas the opposite was true for the same genotypes in never-smokers (**Figure 2**).

It should be noted here, however, that the R^2^ coefficient of determination values for all tested *ERAP1* SNPs were between 0.022 and 0.032 (**Supplementary Table 6**) which means that genotypes of *ERAP1* SNPs in combination with the information about gender and smoking status explain only 2–3% of the variability of the age of onset.

SNPs in other APM genes did not exhibit any associations with age at diagnosis.

#### Stage of the Disease, *ERAP1* and *PSMB9*

Two SNPs revealed associations with the disease stage: *rs27044* in *ERAP1* and *rs17587* in *PSMB9* (**Table 5**). We noticed that the main division for cut-off of age at diagnosis was 62.5 years. Generally, the mean disease stage was lower in patients diagnosed later (*i.e.*, above 62.5 years) and corresponded to value of 2.55 on the severity scale 1–4. In patients who were diagnosed with NSCLC before reaching the age of 62.5 years, the average disease severity was 2.88.

**Table 5:** Stage of NSCLC according to age at diagnosis and *PSMB9 rs17587* and *ERAP1 rs27044* polymorphisms.

Age and SNPs interactions					n,	Stage				N	Mean (SD)		
					%	1	2	3	4				
				**<54.5**	nc %	1 *6.25*	1 *12.5*	8 *62.5*	6 *100%*	16	3.19 (0.83)		
			GA									2.65 (1.11)	
	**<62.5**	*rs17587*		**>54.5**	n %	15 *28.30*	9 *45.28*	17*77.36*	12 *100%*	53	2.49 (1.14)		2.88 (1.11)
			AA or GG		nc %	17 *16.19*	9 *24.76*	32 *55.24*	47 *100%*	105		3.04 (1.1)	
Age at diagnosis													
			CG		nc %	26 *32.10*	18 *54.32*	22 *81.48*	15 *100%*	81		2.32 (1.11)	
	**>62.5**	*rs27044*											2.55 (1.16)
			CC or GG		nc %	30 *24.79*	14 *36.36*	38 *67.77*	39 *100%*	121		2.71 (1.16)	
*D* ^2^ = 22.53; *p* = 0.000299													

**Figure 3:**
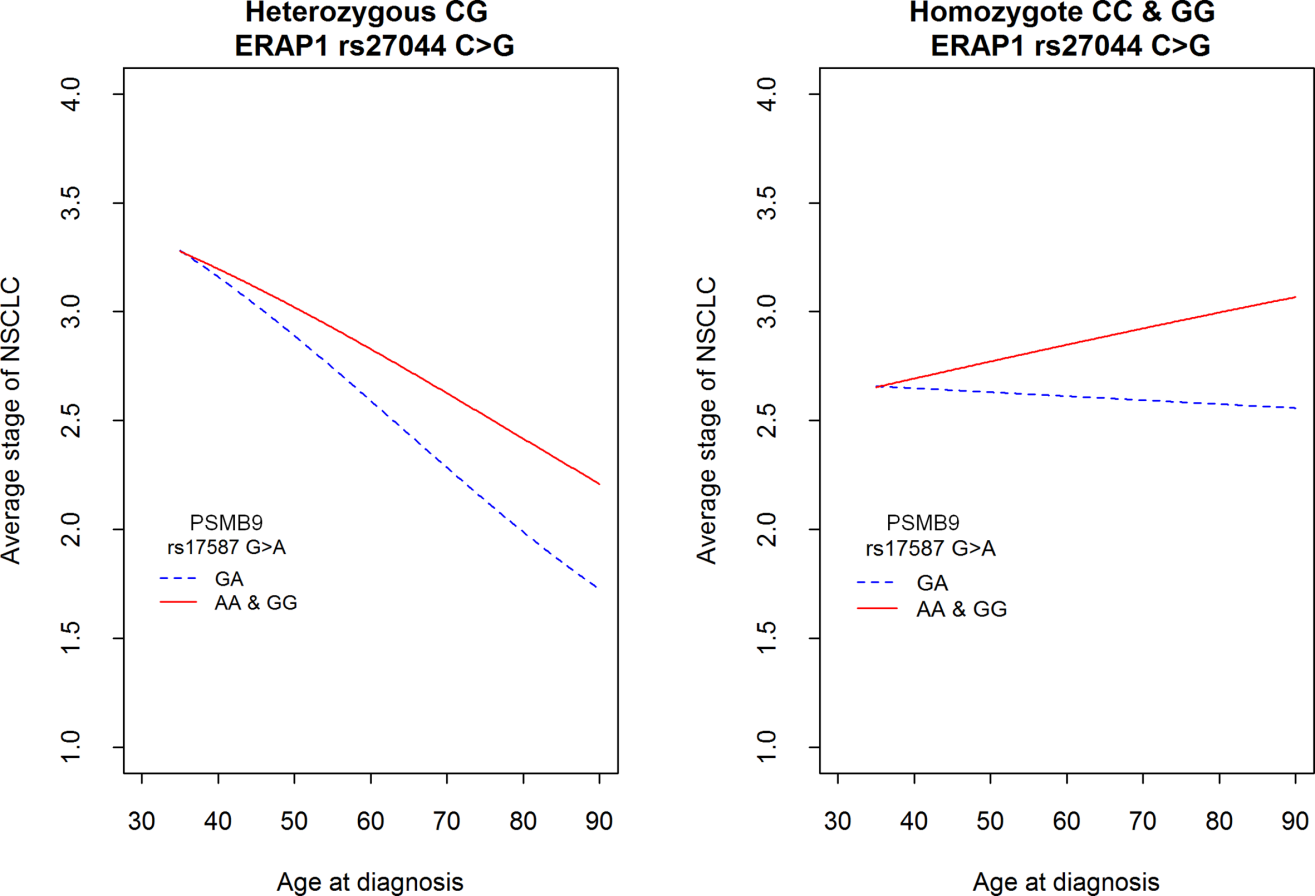
Average stage of NSCLC according to the age at diagnosis and two polymorphisms in genes *PSMB9 rs17587* and *ERAP1 rs27044*. See also **Table 5** for comparison of differences among two SNPs. Hypothesis H0: *There is no difference between genotypes among PSMB9 (red vs. blue line) as well as there is no difference between genotypes of ERAP1* was tested against H1: *This is not true at least in one gene*; *p* = 0.000299.

See also **Figure 3**.

SD, standard deviation.

Additionally, in patients diagnosed before the age of 62.5 years, the value of the mean stage of disease was different depending on the *PSMB9 rs17587* genotype: GA heterozygotes had a lower value (2.65) than both homozygotes (AA and GG, 3.04). When GA heterozygote group was divided into those with age at diagnosis of 54.5 years or less, and those above 54.5 years, then the first group had mean disease stage of 3.19, whereas the second group had only 2.49 (**Table 5**).

On the other hand, in the group of patients diagnosed at an age above 62.5, the mean disease stage depended not on the *PSMB9 rs17587*, but on the *ERAP1 rs27044* genotype. In this case heterozygotes CG had a lower mean disease stage (2.32) than both homozygotes (2.71) (**Table 5**).

**Figure 3** shows a mean expected disease stage in relation to age at diagnosis and both polymorphisms: *PSMB9 rs17587* and *ERAP1 rs27044*. It shows that the mean stage of disease decreased with age at diagnosis, but only in a group of *ERAP1 rs27044* CG heterozygotes (left panel). This decrease was stronger in *PSMB9* heterozygotes than in both homozygotes. In contrast, in a group of both *ERAP1 rs27044* homozygotes the age at diagnosis was not important for disease stage in a group of *PSMB9* heterozygotes, but the mean stage of disease increased with the age at diagnosis in *PSMB9* homozygotes (right panel). Other genetic markers were not associated with the stage of disease. Also gender and smoking did not play a role as factors affecting stage of NSCLC, both when they were considered independently or together with age at diagnosis and genetic polymorphisms (data not shown).

#### Surgery, *ERAP1 rs30187*, and Overall Survival

In this study, NSCLC patient’s OS depended on several factors: disease stage, gender, treatment, and *ERAP1 rs30187* polymorphism.

In our group of patients we observed that the risk of death increased with the severity of disease: with each point on the scale of disease stage this risk increased *HR_stage_* = 1.53 times [CI95% (1.22; 1.92), p = 0.00022]. Therefore, a patient in disease stage III had $HR_{stage}^{III\;vs\;I}=2.34$ times higher death risk than a patient in disease stage I. A patient in the disease stage IV had $HR_{stage}^{IV\;vs\;I}=3.58$ times higher death risk than a patient in stage I.

Generally, men had a higher risk of death than women. At any time from diagnosis, death risk for a man was *HR_man_* = 1.63 higher than that of a woman in the same age, with the same disease stage and the same *ERAP1 rs30187* genotype [CI95% (1.09; 2.43), p = 0.0161].

Surgery was the most important factor for a patient’s survival. When we compare two patients of the same gender, at the same disease stage and the same *rs30187* genotype, then the individual who did not have a surgery had a death risk *HR_surgery_* = 3.88 higher than a person who underwent surgery [CI95% (2.33; 6.48), p = 1.03 × 10^−7^].

Finally, the *ERAP1 rs30187* genotype also affected survival: patients being the carriers of the T allele (TT homozygote or CT heterozygote) had $HR_{\text{rs}30187\text{ C }>T}^{ERAP1}=1.46$ times higher risk of death than patients carrying CC genotype with the same disease stage, gender, age at disease diagnosis, treatment and other features [CI95% (0.82; 2.68), p = 0.0972]. This effect was relatively strong: the death risk for a person with genotype CT or TT in comparison with CC was as high as the death risk of an individual in stage III compared to a patient in stage II (see above).

**Table 6** shows the survival of patients 5, 12, and 21 months after diagnosis depending on surgery as the strongest factor affecting survival, and on *ERAP1 rs30187* genotype. We observed that in the group of patients who underwent surgery, higher percentages of those with *ERAP1 rs30187CC* genotypes survived after 5, 12, and 21 months as compared to those with CT or TT genotype. Interestingly, even in the group of patients who did not qualify for surgery, there was a similar difference between CC *versus* CT + TT genotypes in months 5 and 12. After 21 months the growth of a tumor was so advanced that the *rs30187* genotype had no effect.

**Table 6:** Percent of survivors after three time points: 5, 12, and 21 months after diagnosis according to type of treatment (surgery or not) and polymorphism *rs30187* of the *ERAP1* gene.

Surgery	*ERAP1 rs30187*	n	Time from diagnosis (months)		
			5	12	21
Yes	CC	85	96.4%	89%	80.2%
			CI95(92.6%; 100%)	(82.4; 96)	(71.7; 89.8)
	CT and TT	98	91.8	83.4	69.5
			(86.6; 97.4)	(76.3; 91.2)	(60.6; 79.7)
No	CC	48	66.4	29.1	3.1
			(54.2; 81.3)	(18.2; 46.5)	(0.46; 20.7)
	CT and TT	75	58.3	20.8	9.5%
			(78; 70.7)	(12.7; 34)	CI95(4.18%; 21.3%)

**Figure 4:**
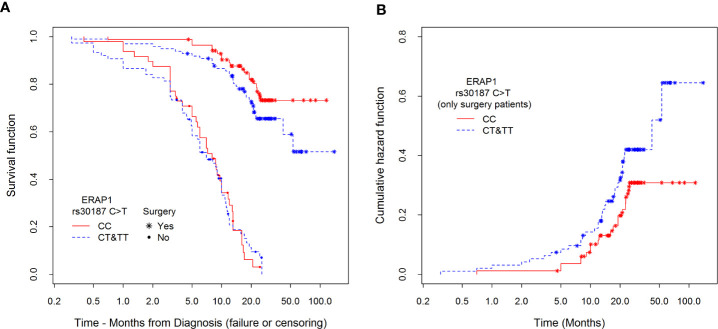
Kaplan–Meier estimators of **(A)** survival functions among patients divided according to type of treatment (surgery or not) and polymorphism *rs30187* in *ERAP1* gene and **(B)** cumulative hazards depending on the polymorphisms only among surgical patients. Hazard risk $HR_{\text{rs}30187\text{C}>T}^{ERAP1}=1.46\;(p=0.0972)$ for differences between CC and CT&TT groups. Estimation of this effect is adjusted to sex, stage of NSCLC, type of treatment, age at diagnosis, and smoking history.

The data for **Table 6** were implemented from survival curves shown in **Figure 4**. In the left panel (A) of the figure survival curves of patients submitted to surgery or those who were not qualified for it, divided according to their *ERAP1 rs30187* genotype are presented. We were able to note, firstly, that survival of non-operated patients was much shorter than those who were subject to surgical intervention. Secondly, survival of CC homozygotes was better than that of patients with CT and TT genotypes. This effect was much stronger for patients who underwent surgery than for non-operated patients; importantly, the effect was detectable even for the latter ones during the first three months of observation.

The right panel (B) presents an estimation of cumulative hazard function (only for patients who underwent surgery) confirming previously described relationship between the possession of CT or TT genotype and higher risk of death in comparison to CC genotype during the whole observation period.

#### Response to Chemotherapy and *ERAP1 rs27044*

Only one SNP, *rs27044* in *ERAP1*, was associated with response to chemotherapy. Namely, a heterozygous CG genotype was associated with better response, whereas both homozygotes responded worse (**Table 7**). That is, only 20.0% of heterozygotes (CG), but 30.12% of homozygotes (CC and GG), had tumor progression despite chemotherapy. Complete response was achieved by 26.7% of heterozygotes but only 16.87% of homozygotes. Therefore, a heterozygote had OR = 1.76 times higher chance to respond better to chemotherapy than had a homozygote (p = 0.0698) adjusted for disease stage, smoking status, age at diagnosis, gender, and the same surgery past (*i.e.*, yes or not).

**Table 7:** Response to chemotherapy depending on *ERAP1 rs27044* polymorphism and adjusted by clinical characteristics listed below.

ERAP1rs27044	n,%	Progressive disease	Stable disease	Partial response	Complete response
CG	45	9	7	17	12
	%	20.0	15.6	37.8	26.7
	cum.%	20.0	35.6	73.3	100.0
CC and GG	83	25	12	32	14
	%	30.12	14.46	38.55	16.87
	cum.%	30.1	44.6	83.1	100.0
OR=1.76, CI95(0.92;3.33); p=0.0698^1^					

^1^—effect adjusted to the type of treatment (surgery or not), gender, stage of NSCLC, age at diagnosis and smoking history.

## Discussion

Gene expression profiles of cancers arising in smokers and never smokers are very different (8, 46, 47). Also mutations in lung cancer cells differ in smokers and never-smokers; for example, those in *TP53* and *KRAS* genes are more frequent in smokers, whereas lung cancer in never smokers is characterized by *EGFR* TK mutations, *ALK*, *RET* and *ROS* fusions. Mutation numbers are also lower in never-smokers, but most of them are suspected to be causative for malignant transformation, whereas mutations in smokers, although more numerous, are believed to be mostly passengers without effect on transformation (48). Moreover, it has been shown that multiple germ-line genetic polymorphisms affect susceptibility to lung cancer differently in smokers and never-smokers. Thus, the effects of polymorphisms in DNA repair genes were found to depend on smoking status: for example, polymorphisms of *XRCC1* and *ERCC2*, involved in decreased adduct removal, were associated with risk in never or light smokers, but tended to be protective in heavy smokers. The authors explain this paradoxical finding as follows: “Although the reasons for these differences are not clear, it is possible that in heavy smokers, the effects of the DNA repair polymorphisms are overwhelmed” (8). Therefore, lung cancer in smokers and never-smokers are, according to many authors, two different diseases (49). It is conceivable, therefore, that genetic associations of APM polymorphisms to NSCLC in smokers (where tobacco smoke is the strongest factor) and never-smokers (where genetics may play much stronger role in addition to environmental factors other than tobacco) may differ.

The main issues addressed in our study were: (i) whether smokers and never-smokers differ in the associations of APM polymorphisms with NSCLC risk and clinical outcome, (ii) whether *ERAP2* influences the *ERAP1* effect, if any, as we observed it in other diseases—psoriasis (50), and ankylosing spondylitis (51).

In this study we demonstrated that all tested *ERAP1* single nucleotide polymorphisms, except *rs26618*, conferred susceptibility to cancer not only in never-smokers, but also in smokers, although in the opposite direction. Therefore, as far as we know, we describe here for the first time the opposite associations of *ERAP1* polymorphisms with NSCLC in smokers *versus* never-smokers. This result may be explained by the differing expression of distinct proteins as well as their different mutations in smokers *versus* never-smokers. This should result in differences in repertoire of neoantigenic peptides bound and presented by class I HLA molecules between smokers and never-smokers. Different peptides, in turn, may require different ERAP variants for efficient antigen presentation to cytotoxic T cells, resulting in elimination (or not) of cancer cells. Of course, multiple differences in peptide repertoire may exist also at the level of individuals in each of these groups, and some of them may blur the differences between smokers and never-smokers to some extent. Hence the detectable effects of individual SNPs and especially their haplotypes (in which the same allele of an individual SNP may be distributed in many haplotypes) were not very strong.

The *rs2248374* in *ERAP2* was not associated with susceptibility to NSCLC when analyzed individually, however we obtained interesting results for this variant when it was analyzed as a part of the haplotype with *ERAP1* SNPs. Normal ERAP2 protein may likely form not only ERAP2 homodimers (52) but also ERAP2/ERAP1 heterodimers in which both enzymes may be functional and, in some cases, both of them are required to produce some epitopes (45). In light of this, we found the result of our analysis for *rs26653G-rs26618C-rs2287987T-rs30187C-rs27044C-rs2248374* ***G*** [ERAP1 127P/276M/349M/528R/730E, ERAP2(**-**)] and *rs26653G-rs26618C-rs2287987T-rs30187C-rs27044C-rs2248374* ***A*** [ERAP1 127P/276M/349M/528R/730E, ERAP2(**+**)] haplotypes intriguing. Both haplotypes contain the same *ERAP1* alleles, and in both of them the combination of these alleles should probably result in low enzymatic activity (because of possessing Arg528 and Glu730 residues) (53–55). The former haplotype, ERAP2-negative, was protective (albeit non-significantly) in smokers, but it was associated with higher risk in never-smokers. The latter haplotype with the same *ERAP1*, but differing only by the presence of functional ERAP2, did not differentiate between smokers and never-smokers. *rs2248374A* allele gives normal ERAP2 protein expression, whereas *rs2248374G* produces unstable mRNA degraded by nonsense mediated decay and usually results in undetectable protein expression (56). However, it has been reported recently that during stimulation by microbial infections the *rs2248374G* allele may give two truncated protein isoforms absent in *rs2248374AA* homozygotes and with an unknown biological role (57–59), possibly interfering in ERAP1 activity. Why the haplotype GCTCC**G** without functional ERAP2 molecule gives opposite results in never-smokers *versus* smokers, whereas the same ERAP1 (GCTCC**A**) with functional ERAP2 does not affect NSCLC prevalence in any of these groups, is not clear. As mentioned earlier, smokers and never-smokers suffering from lung cancer differ in gene expression, germ-line genetic polymorphisms, and total number of mutations (8, 46–48). Therefore, we may speculate that in smokers the GCTCC**G** haplotype results in production of epitopes which could stimulate cytotoxic T cells to kill cancer cells. In never-smokers, with the same *ERAP1* haplotype, other proteins are expressed being potential source of epitopes leading to the recognition and killing of cancer cells by T cells, but this particular ERAP1 variant does not produce suitable epitopes from them. This may result in uncontrolled growth of the tumor. The GCTCC**A** haplotype, coding for active ERAP2, allows this enzyme, either alone or in a heterodimer with ERAP1 to destroy stimulating epitopes in never-smokers and protective epitopes in smokers, resulting in a lack of ERAP effect on NSCLC risk.

Interestingly, in never-smokers we observed also that the haplotype *rs26653G-rs26618T-rs2287987C-rs30187C-rs27044C-rs2248374* ***G*** [ERAP1 127P/276I/349V/528R/730E, ERAP2(-)] significantly increased the risk of NSCLC, whereas the haplotype *rs26653C-rs26618T-rs2287987T-rs30187T-rs27044G-rs2248374* ***G*** [ERAP1 127R/276I/349M/528K/730Q, ERAP2(−)] decreased the risk. Both haplotypes encode an inactive variant of ERAP2, however, the former encodes ERAP1 residues (528R, 730E) associated with low enzymatic activity, but the latter codes for residues (528K, 730Q) associated with high activity (55). Then, a combination of low activity ERAP1 and the lack of an active form of ERAP2 seems once again to favor the disease in never-smokers and this is a similar situation to that described above for haplotype *rs26653G-rs26618C-rs2287987T-rs30187C-rs27044C-rs2248374G*. On the other hand, highly active ERAP1 without the presence of functional ERAP2, may appear to be suitable to produce cancer epitopes effectively recognized by immune cells capable of eliminating cancer cells.

It is important to mention here that the effect of ERAPs on tumor antigen presentation depends mainly on HLA-I allotypes which are highly polymorphic, and therefore distinct in different individuals. Hence, the influence of the ERAPs without stratification for HLA-I alleles cannot be very strong. In addition, not all epitopes to be bound by HLA-I and presented to T cells require trimming by ERAPs (60).

It should be explained here why we have not detected earlier any association of *ERAP1* SNPs with NSCLC in Poles, whereas they were found in Chinese populations (40). In that work, we had incomplete information on the smoking status of patients and controls, therefore we could not use it in statistical calculations. As we see here, *ERAP1* SNPs seem to act in opposite directions in smokers and never-smokers. Therefore, in a randomly selected representation of Polish populations, consisting of smokers and never-smokers, these effects mutually neutralized themselves. In Chinese populations examined in that study smokers might have accounted for a smaller proportion of tested individuals (Dr. Yao Yufeng, personal communication), and indeed, their results for *rs26653* were concordant with those of our never-smokers, although for *rs27044* they were concordant rather with our smokers, and inconsistent with our results for other *ERAP1* SNPs independently of smoking status [compare our **Table 2** with Table 1 of Yao et al. (40)]. However, Chinese people are genetically distant from Poles, as reflected in highly significant differences of *ERAP1* SNP frequencies in healthy Chinese and Polish populations [(40), **Table 2**] as well as *HLA-I* allele frequencies [www.allelefrequencies.net] which play a major role in presentation of tumor antigens. Interestingly, *ERAP2* SNPs were found to be associated with NSCLC in the Chinese population even without stratification for smoking status as compared to healthy individuals, and there were also differences between smoking and never-smoking patients. However, these results were published in Chinese only (61), so their verification on the basis of short English abstract is not possible. Nevertheless, it looks as if both *ERAPs* are associated with NSCLC in Chinese even without division into smokers and never-smokers. For *ERAP2*, like for *ERAP1*, this may be explained by differences in allele frequencies for these variants between Chinese and Polish populations. Indeed, frequencies of *rs2248374* A and G alleles were 37.8 and 62.2%, respectively, in healthy Chinese individuals (61), but 49.1 and 50.9% in Poles (our data).

In this work, we revealed interesting associations of some APM polymorphisms with: age at diagnosis (*ERAP1 rs26653*), disease stage (*ERAP1 rs27044*, *PSMB9 rs17587*), overall survival (*ERAP1 rs30187*), and response to chemotherapy (*ERAP1 rs27044*).

We demonstrated that some *ERAP1* genotypes which exerted NSCLC risk or protection also influenced the patient’s age at diagnosis. We observed an interesting relationship: the higher disease risk a given genotype bears, the lower AAD (earlier diagnosis) is expected, and it was seen most clearly for *rs26653*. In smokers, *rs26653CC* homozygosity predisposed to NSCLC and decreased AAD by about 3 years as well. In contrast, in never-smokers the same genotype protected against lung cancer and increased the age at diagnosis by about as much as 10 years in comparison to GG and GC genotypes. We may try to explain it by the supposition that *rs26653CC* never-smoking subjects develop cancer more seldom than others and are diagnosed significantly later because they suffer from a more benign lung tumor. Similar, but weaker associations were observed for *rs30187*, where, as we expected in never-smokers, the protective genotype TT also elevated patient’s age of diagnosis. In smokers, vice versa, the TT genotype was weakly predisposing to cancer, and in line with that, decreasing the age at diagnosis.

There is no information in literature about a similar association of any known genetic variant with age of diagnosis of NSCLC, therefore these observations need further development. Interestingly, *rs26653* in *ERAP1* has been associated with several autoimmune diseases including psoriasis (62–64), ankylosing spondylitis and inflammatory bowel disease (65). This information indicates that *rs26653* may be functional. However, data regarding its potential impact on enzyme activity or expression level are scarce. The *rs26653* heterozygote was claimed by Mehta et al. (66) to be associated with normal ERAP1 expression in cervical carcinoma when contrasted with both homozygotes which had lower expression (in terms of proportion of positive cells in immunohistochemistry). However, no detailed data were shown. They also demonstrated for the same cancer that *rs26653* heterozygotes had better survival than both homozygotes (66). In an earlier report, the same authors showed that low ERAP1 expression was significantly associated with reduced survival of patients with cervical carcinoma (67). However, this SNP had no effect on the survival of our patients with NSCLC, in contrast to *rs30187* (discussed below). For the role of ERAP1 expression in other cancers, see (29). It should be noted that overall influence of *ERAP1* genotypes on AAD was relatively small. Even in the case of *rs26653* (where association was the strongest) the effect of this polymorphism in combination with additional information about smoking status and patient’s gender explains only 3.2% of the entire variability of AAD.

Our results indicated that the stage of cancer was primarily associated with age at diagnosis: the mean disease stage was lower in patients diagnosed for NSCLC at a later age. We may explain it by the assumption that a less malignant, slower growing tumor causes symptoms prompting the patient to see a doctor for the first time later than a more malignant, fast-growing cancer, hence the later age of diagnosis correlates with lower disease stage.

The influence of SNPs on cancer stage was observed here for the *rs27044* in *ERAP1* and *rs17587* in *PSMB9*. For both SNPs, the heterozygotes had lower stage of disease, particularly in later age. Interestingly, the decline of disease stage together with the increasing AAD was the strongest in those patients who were simultaneously heterozygotic for both *rs27044* and *rs17587*. In contrast, in patients homozygotic for *rs27044C* or *27044G* and simultaneously homozygotic for *rs17587A* or *rs17587G*, the disease stage was growing with age at diagnosis, while in *rs17587* heterozygotes but homozygotic for *rs27044C* or *27044G*, the stage of cancer did not change with age. In all these situations, the effect of *rs27044* was stronger than that of *rs17587*, suggesting that a difference in the production of peptides by different variants of *PSMB9* is for disease stage less important than more or less active trimming of these peptides by ERAP1, depending on *rs27044*. For *rs17587*, it was reported that in GG individuals, the immunoproteasome activity was higher than in GA individuals (68), but this result was not confirmed subsequently in a large panel of cancer cell lines (69). Therefore, the difference in activity of two *PSMB9* variants seems to be weak if any, hence the stronger effect of ERAP1 variant. In addition, as *rs17587* did not affect susceptibility to lung cancer, we cannot expect its strong contribution to the staging of cancer. Some effects of *rs17587* heterozygosity, modulating to some extent the *rs27044* effect, might result from a wider spectrum of peptides produced by double heterozygotes.

We showed that NSCLC patient’s overall survival mostly depended on implemented surgery. As anticipated, non-operated patients had death risk (at any time) about four-fold higher than operated patients. Other factors influencing OS were: stage of cancer, gender, and, unexpectedly, the *ERAP1 rs30187* genotype. Patients with CC genotype in this polymorphism survived significantly longer than patients possessing CT or TT. Of note, the effect of the *rs30187* SNP was seen both in operated and non-operated patients.

Moreover, the effect of *rs30187* on survival was relatively strong, particularly in patients operated upon, which suggests the influence of the *rs30187* variation on the repertoire of peptides contributing to immune-mediated elimination of residual cancer cells. Therefore, *rs30187* in *ERAP1* was found here to be associated both with risk of cancer and survival. The *rs30187* SNP is located in hinge domain III and can indirectly affect enzymatic function by affecting the conformational dynamics of ERAP1 (70). In addition, as mentioned previously, the rs30187C allele is a part of several *ERAP1* haplotypes (71) coding for a low activity form of the enzyme (53). If CC homozygotic patients suffering from NSCLC survived longer, we may suppose that less active ERAP1 favored generation of much more immunogenic cancer epitopes recognized by the immune system. Alternatively, high-activity ERAP1 present in patients with TT and, at lower level, with CT genotypes might destroy (by over-trimming) some cancer epitopes, and therefore the efficient cancer elimination by immune cells was impaired. Then, we suppose that in patients with *rs30187CC* genotype, effective treatment along with more efficiently working immune system are able to prolong their life.

To date there was no published data about the influence of *ERAP1* SNPs on survival of NSCLC patients. In a single study concerning cervical carcinoma, Mehta and colleagues analyzed the influence of several SNPs in *ERAP1* (including *rs30187*) on disease risk and OS. However, the association with OS was found only for *rs3734016* and *rs26653* but not for *rs30187* (66). Thus, *ERAP1* polymorphisms may work differently in response to different cancers most likely associated with different HLA alleles and therefore requiring different preparation of antigenic peptides for efficient immune response.

In response to chemotherapy, only one SNP, *ERAP1 rs27044*, seemed to play some role. Here, again, the heterozygote had an advantage over both homozygotes: complete response was achieved by 26.7% of patients with CG genotype, whereas only 16.9% of homozygotes (CC and GG) reached this result. Similarly, progressive disease in spite of therapy was observed in only 20.0% of heterozygotes but in 30.1% of homozygotes. The observed effect concerned patients with either a complete response or, in contrary, with disease progression, but was undetectable in patients who experienced partial response or stable disease. It is unclear why patients heterozygous for *rs27044* had some advantage over those with homozygous genotypes. In contrast to *ERAP1 rs30187* [R528K] that directly influences the kinetics of the enzyme, *rs27044* [E730Q] may modulate enzymatic activity depending on substrate length. The presence of glutamic acid in 730 position increases preference of ERAP1 for shorter peptides (37, 70). Thus, we suggest that in the heterozygotes both allotypes of ERAP1 could in total utilize a broader spectrum of substrates and the chance of generation of more immunogenic cancer epitope(s) is much greater than in the case of homozygotes. In this scenario “useful” cancer epitopes could be produced efficiently by both alleles. However, this finding requires confirmation on a larger cohort of patients and, optimally, also *in vitro* study on peptide trimming efficiency of different ERAP1 allotypes, as we have made for other SNP in other disease (72). If confirmed, it may appear useful as a predictor of a better treatment response.

It should be mentioned here that advantage (or disadvantage) of heterozygote over both homozygotes (*i.e.*, positive or negative heterosis) has been observed in many clinical situations, the best known example is the advantage of sickle cell anemia heterozygotes in regions where malaria is prevalent: wild-type homozygotes are prone to malaria, sickle cell homozygotes are prone to anemia, and heterozygotes are the best survivors (73).

One of the limitations of the current study is an insufficient number of never-smoking participants, especially among NSCLC patients. This resulted from the dominant contribution of smoking to lung cancer prevalence.

## Conclusions

To the best of our knowledge, this is the first study which discovered the adverse associations of *ERAP1* polymorphisms with NSCLC in smokers and never-smokers. We described also the relationships between some tested APM variants (again, mostly in *ERAP1*) and several clinical parameters such as age at diagnosis, disease stage, overall survival and response to chemotherapy. Multiple associations of *ERAP1* genetic variants, not only with NSCLC risk but also with disease course and response to treatment, described here, support the thesis that ERAP1 aminopeptidase can take part in the anti-cancer immune response. Although *rs2248374* in *ERAP2*, analyzed separately, was not associated with NSCLC, the presence or absence of functional ERAP2 in a few haplotypes seems to modify the effect of ERAP1 to some extent, especially in the case of the ERAP1 variant with low enzymatic activity. Our novel results, however, need to be considered as preliminary and therefore further developed and confirmed by other research groups. It is worth studying, as its results may contribute to better prediction of treatment outcomes.

## Data Availability Statement

The original contributions presented in the study are included in the article/**Supplementary Material**, further inquiries can be directed to the corresponding author.

## Ethics Statement

The studies involving human participants were reviewed and approved by the Bioethical Committee of Wrocław Medical University, Wrocław, Poland. The patients/participants provided their written informed consent to participate in this study.

## Author Contributions

PK and AW conceived and designed the experiments. AW, MJ, MW, and WN-M performed the experiments. MS performed statistical analysis. KP, IP, and AK contributed to patients’ recruitment and clinical characteristics. JD and NJ contributed to controls recruitment. PK, AW, MS, and IP wrote the paper. MJ and MW critically reviewed the paper. All authors contributed to the article and approved the submitted version.

## Funding

This work was supported by a grant OPUS 8 2014/15/B/NZ5/03517 from the Polish National Science Centre.

## Conflict of Interest

The authors declare that the research was conducted in the absence of any commercial or financial relationships that could be construed as a potential conflict of interest.
