# Supplementary material for: Polymorphisms of Antigen-Presenting Machinery Genes in Non-Small Cell Lung Cancer: Different Impact on Disease Risk and Clinical Parameters in Smokers and Never-Smokers
Source: Front Immunol. 2021 May 31;12:664474. doi: 10.3389/fimmu.2021.664474 (PMC8212834; doi:10.3389/fimmu.2021.664474)

Supplementary Material

**Supplementary Table 1.** Characteristics of control group

| Age^1^ | **Gender** | **Min** | **Q1** | **Median** | **Sn** | **Q3** | **Max** | **n** |
| --- | --- | --- | --- | --- | --- | --- | --- | --- |
|  | Men | 22 | 42 | 63 | 13 | 69 | 84 | 367 |
|  | Women | 23 | 35 | 53 | 16 | 63 | 82 | 42 |

^1^ – age at the moment of blood sampling; Q1, Q3 – first and third quartile; Sn – measure of variability.

**Supplementary Table 2.** Smoking history among control men and women according to the current status and the availability of data.

| **Smoking** | **Smokers** | | **Never-smokers** | | **Smoking history**  **unknown** | | **∑** |
| --- | --- | --- | --- | --- | --- | --- | --- |
| **Gender** | **n** | **%** | **n** | **%** | **n** | **%** | **n** |
| **Men** | 161 | 43.9 | 145 | 39.5 | 61 | 16.6 | 367 |
| **Women** | 12 | 28.6 | 10 | 23.8 | 20 | 47.6 | 42 |

**Supplementary Table 3**. Characteristics of the SNPs

| **Chr.** | **Gene** | **HGNC ID for gene** | **SNP** | **DNA Strand** | **Variation** | **Assay ID** |
| --- | --- | --- | --- | --- | --- | --- |
| 5 | *ERAP1* | 18173 | *rs26653* | Fwd | G>**C**, P127**R** | C__794818_30 |
| 5 | *ERAP1* |  | *rs26618* | Fwd | T>**C**, I276**M** | C__3056894_10 |
| 5 | *ERAP1* |  | *rs2287987* | Fwd | T>**C**, M349**V** | C__3056893_20 |
| 5 | *ERAP1* |  | *rs30187* | Fwd | C>**T**, R528**K** | C__3056885_10 |
| 5 | *ERAP1* |  | *rs27044* | Fwd | C>**G**, E730**Q** | C__3056870_10 |
| 5 | *ERAP2* | 29499 | *rs2248374* | Fwd | G>**A**, intron | C__25649529_10 |
| 6 | *TAP1* | 43 | *rs1135216* | Rev. | T>**C**, D697**G** | C__531909_20 |
| 6 | *TAP1* |  | *rs1057141* | Rev. | T>**C**, I393**V** | C__549926_20 |
| 6 | *TAP2* | 44 | *rs4148876* | Rev. | G>**A**, R651**C** | C_30159972_10 |
| 6 | *TAP2* |  | *rs1800454* | Rev. | C>**T**, V379**I** | C__8848961_20 |
| 6 | *TAP2* |  | *rs241447* | Rev. | T>**C**, T665**A** | C_175701925_10 |
| 6 | *TAP2* |  | *rs16870908* | Fwd | G>**A**, L647**F** | C_34171660_10 |
| 6 | *PSMB9* | 9546 | *rs1351383* | Rev. | A>**C**, intron | C_8848996_10 |
| 6 | *PSMB9* |  | *rs2127675* | Rev. | A>**G**, intergenic† | C_15827453_10 |
| 6 | *PSMB9* |  | *rs17587* | Fwd | G>**A**, R60**H** | C_8849004_1 |
| 6 | *PSMB8* | 9545 | *rs2071543* | Rev. | G>**T**, Q49**K** | C_15869253_10 |

Minor alleles detected in the Polish population were **bolded**. Fwd - forward, Rev. – reverse.

†In close proximity to *PSMB9*.

**Supplementary Table 4.** Power of the chi-square test for association between genotype and risk of cancer. Assumptions: significance level $\alpha=0.05$, strata: smoking, $N_{Caces}=444$, $N_{Controls}=329$, additive model of the risk, i.e. $\ln\frac{\pi_{YY}}{1-\pi_{YY}}-\ln\frac{\pi_{XX}}{1-\pi_{XX}}=2\left[ \ln\frac{\pi_{XY}}{1-\pi_{XY}}-\ln\frac{\pi_{XX}}{1-\pi_{XX}} \right]$, where $\pi_{YY}, \pi_{XY}, \pi_{XX}$ is probability of cancer for individual with genotype $XX, XY, YY$, respectevitelly. Genotype $XX$ as baseline. Null hypothesis H0: *OR.XY/XX = OR.YY/XX = 1 both in smoking and never-smoking group*; alternative H1: *Risk increases with number of allels Y*. Power is probability (x100%) of rejection of a H0 depending on the strength of the association measured with ORs. Chi-square test on 2 degrees of freedom.

| **OR.XY/XX** | **OR.YY/XX** | **Power (%)** |
| --- | --- | --- |
| 1.00 | 1.00 | 5.03 |
| 1.03 | 1.06 | 5.15 |
| 1.06 | 1.11 | 7.10 |
| 1.08 | 1.18 | 9.60 |
| 1.11 | 1.24 | 13.43 |
| 1.14 | 1.30 | 17.40 |
| 1.17 | 1.37 | 23.43 |
| 1.20 | 1.44 | 31.35 |
| 1.23 | 1.51 | 38.30 |
| 1.26 | 1.58 | 45.58 |
| 1.29 | 1.65 | 55.78 |
| 1.32 | 1.73 | 62.78 |
| 1.34 | 1.81 | 69.75 |
| 1.37 | 1.89 | 77.00 |
| 1.40 | 1.97 | 82.73 |
| 1.43 | 2.06 | 86.70 |
| 1.47 | 2.15 | 90.50 |
| 1.50 | 2.24 | 92.98 |
| 1.53 | 2.33 | 95.78 |
| 1.56 | 2.42 | 96.83 |
| 1.59 | 2.52 | 98.13 |
| 1.62 | 2.62 | 98.50 |
| 1.65 | 2.73 | 99.28 |
| 1.68 | 2.83 | 99.30 |
| 1.71 | 2.94 | 99.75 |
| 1.75 | 3.05 | 99.88 |
| 1.78 | 3.16 | 99.93 |
| 1.81 | 3.28 | 99.98 |
| 1.84 | 3.40 | 99.99 |
| 1.88 | 3.52 | 100.00 |

**Supplementary Table 5**. *TAP1, TAP2, PSMB9* and *PSMB8* genotype distributions among NSCLC patients and controls and conditional association between SNP genotype and cancer after adjusting for smoking as the stratification variable.

| **Gene** | **SNP** | **Smoking** | **n, %** | **Patients** | | | | **Controls** | | | **OR**  (CI95%) | | **OR.MH**  (CI95%) | | ***P**** |
| --- | --- | --- | --- | --- | --- | --- | --- | --- | --- | --- | --- | --- | --- | --- | --- |
| *TAP1* | *rs1135216* |  | n, % | **CC** | | **TC** | **TT** | **CC** | **TC** | **TT** | **TC/**  **TT** | **CC/**  **TT** | **TC/**  **TT** | **CC/**  **TT** | 0.647 |
|  |  | smokers | n | 4 | | 80 | 292 | 1 | 39 | 132 | **0.92**  (0.61; 1.46) | **1.36**  (0.26; 7.15) | **0.78**  (0.54; 1.14) | **1.19**  (0.36; 4.24) |  |
|  |  |  | % | *1.1* | | *21.3* | *77.7* | *0.6* | *22.7* | *76.7* |  |  |  |  |  |
|  |  | never-  smokers | n | 1 | | 6 | 49 | 3 | 36 | 117 | **0.42**  (0.14; 0.93) | **1.02**  (0.18; 7.30) |  |  |  |
|  |  |  | % | *1.8* | | *10.7* | *87.5* | *1.9* | *23.1* | *75.0* |  |  |  |  |  |
|  |  | HWE (p-val, f) | | | S(p=0.565; f=-0.03); NS(p=0.150; f=0.3) | | | S(p=0.295; f=-0.08); NS(p=0.904; f=0.01) | | | ${OR}_{smokers}\approx{OR}_{never-smokers}$,  $p_{MH}$ =0.293 | | | |  |
| *TAP1* | *rs1057141* |  | n, % | **CC** | | **TC** | **TT** | **CC** | **TC** | **TT** | **TC/**  **TT** | **CC/**  **TT** | **TC/**  **TT** | **CC/**  **TT** | 0.723 |
|  |  | smokers | n | 10 | | 97 | 269 | 5 | 43 | 124 | **1.04**  (0.69; 3.2) | **0.88**  (0.33; 3.2) | **0.92**  (0.65; 1.31) | **1**  (0.42; 2.64) |  |
|  |  |  | % | *2.7* | | *25.8* | *71.5* | *2.9* | *25.0* | *72.1* |  |  |  |  |  |
|  |  | never-  smokers | n | 2 | | 12 | 43 | 4 | 46 | 106 | **0.66**  (0.3; 1.29) | **1.36**  (0.18; 6.88) |  |  |  |
|  |  |  | % | *3.5* | | *21.1* | *75.4* | *2.6* | *29.5* | *67.9* |  |  |  |  |  |
|  |  | HWE (p-val, f) | | | S(p=0.725; f=0.02); NS(p=0.362; f=0.13) | | | S(p=0.590; f=0.04); NS(p=0.717; f=-0.03) | | | ${OR}_{smokers}\approx{OR}_{never-smokers}$,  $p_{MH}$ =0.508 | | | |  |
| *TAP2* | *rs4148876* |  | n, % | **AA** | | **GA** | **GG** | **AA** | **GA** | **GG** | **GA/**  **GG** | **AA/**  **GG** | **GA/**  **GG** | **AA/**  **GG** | 0.119 |
|  |  | smokers | n | 1 | | 35 | 329 | 0 | 17 | 156 | **0.96**  (0.54; 1.81) | **1.42**  (0.45; 3.48) | **1.34**  (0.82; 2.28) | **1.74**  (0.44; 5.46) |  |
|  |  |  | % | *0.3* | | *9.6* | *90.1* | *0.0* | *9.8* | *90.2* |  |  |  |  |  |
|  |  | never-  smokers | n | 1 | | 12 | 43 | 2 | 15 | 139 | **2.59**  (1.1; 6.01) | **1.92**  (0.33; 16.29) |  |  |  |
|  |  |  | % | *1.8* | | *21.4* | *76.8* | *1.3* | *9.6* | *89.1* |  |  |  |  |  |
|  |  | HWE (p-val, f) | | | S(p=0.920; f=0.00); NS(p=0.901; f=0.02) | | | S(p=0.476; f=-0.05); NS(p=0.042; f=0.16) | | | ${OR}_{smokers}\approx{OR}_{never-smokers}$,  $p_{MH}$ = 0.157 | | | |  |
| *TAP2* | *rs1800454* |  | n, % | **TT** | | **CT** | **CC** | **TT** | **CT** | **CC** | **CT/**  **CC** | **TT/**  **CC** | **CT/**  **CC** | **TT/**  **CC** | 0.304 |
|  |  | smokers | n | 7 | | 93 | 280 | 4 | 34 | 134 | **1.3**  (0.86; 2.08) | **0.8**  (0.24; 3.47) | **1.3**  (0.91; 1.9) | **0.76**  (0.29; 2.45) |  |
|  |  |  | % | *1.8* | | *24.5* | *73.7* | *2.3* | *19.8* | *77.9* |  |  |  |  |  |
|  |  | never-  smokers | n | 0 | | 16 | 41 | 2 | 36 | 118 | **1.29**  (0.62; 2.5) | **0.57**  (0.24; 3.13) |  |  |  |
|  |  |  | % | *0.0* | | *28.1* | *71.9* | *1.3* | *23.1* | *75.6* |  |  |  |  |  |
|  |  | HWE (p-val, f) | | | S(p=0.822; f=-0.01); NS(p=0.177; f=-0.16) | | | S(p=0.303; f=0.08); NS(p=0.695; f=-0.03) | | | ${OR}_{smokers}\approx{OR}_{never-smokers}$,  $p_{MH}$ = 0.980 | | | |  |
| *TAP2* | *rs241447* |  | n, % | **CC** | | **TC** | **TT** | **CC** | **TC** | **TT** | **TC/**  **TT** | **CC/**  **TT** | **TC/**  **TT** | **CC/**  **TT** | 0.983 |
|  |  | smokers | n | 40 | | 161 | 181 | 20 | 71 | 82 | **1.03**  (0.70; 1.51) | **0.9**  (0.51; 1.67) | **0.96**  (0.69; 1.33) | **1**  (0.61; 1.66) |  |
|  |  |  | % | *10.5* | | *42.1* | *47.4* | *11.6* | *41.0* | *47.4* |  |  |  |  |  |
|  |  | never-  smokers | n | 8 | | 18 | 31 | 16 | 60 | 80 | **0.78**  (0.39; 1.53) | **1.32**  (0.46; 3.35) |  |  |  |
|  |  |  | % | *14.0* | | *31.6* | *54.4* | *10.3* | *38.5* | *51.3* |  |  |  |  |  |
|  |  | HWE (p-val, f) | | | S(p=0.647; f=0.02); NS(p=0.061; f=0.25) | | | S(p=0.437; f=0.06); NS(p=0.352; f=0.08) | | | ${OR}_{smokers}\approx{OR}_{never-smokers}$,  $p_{MH}$ = 0.620 | | | |  |
| *TAP2* | *rs16870908* |  | n, % | **AA** | | **GA** | **GG** | **AA** | **GA** | **GG** | **GA/**  **GG** | **AA/**  **GG** | **GA/**  **GG** | **AA/**  **GG** | 0.044 |
|  |  | smokers | n | 1 | | 43 | 312 | 0 | 12 | 161 | **1.8**  (0.99; 3.87) | **1.55**  (0.49; 3.75) | **1.23**  (0.76; 2.06) | **1.84**  (1.08; 3.42) |  |
|  |  |  | % | *0.3* | | *12.1* | *87.6* | *0.0* | *6.9* | *93.1* |  |  |  |  |  |
|  |  | never-  smokers | n | 0 | | 3 | 54 | 0 | 20 | 136 | **0.43**  (0.06; 1.11) | **2.5**  (2.31; 2.73) |  |  |  |
|  |  |  | % | *0.0* | | *5.3* | *94.7* | *0.0* | *12.8* | *87.2* |  |  |  |  |  |
|  |  | HWE (p-val, f) | | | S(p=0.698; f=-0.02); NS(p=0.838; f=-0.03) | | | S(p=0.491; f=-0.04); NS(p=0.342; f=-0.07) | | | ${OR}_{smokers}\approx{OR}_{never-smokers}$,  $p_{MH}$ = 0.097 | | | |  |
| *PSMB9* | *rs1351383* |  | n, % | **CC** | | **AC** | **AA** | **CC** | **AC** | **AA** | **AC/**  **AA** | **CC/**  **AA** | **AC/**  **AA** | **CC/**  **AA** | 0.996 |
|  |  | smokers | n | 70 | | 174 | 125 | 35 | 73 | 60 | **1.14**  (0.75; 1.73) | **0.96**  (0.58; 1.63) | **1.18**  (0.82; 1.68) | **0.95**  (0.61; 1.49) |  |
|  |  |  | % | *19.0* | | *47.2* | *33.9* | *20.8* | *43.5* | *35.7* |  |  |  |  |  |
|  |  | never-  smokers | n | 7 | | 30 | 19 | 24 | 73 | 59 | **1.27**  (0.66; 2.57) | **0.93**  (0.32; 2.36) |  |  |  |
|  |  |  | % | *12.5* | | *53.6* | *33.9* | *15.4* | *46.8* | *37.8* |  |  |  |  |  |
|  |  | HWE (p-val, f) | | | S(p=0.488; f=0.04); NS(p=0.36; f=-0.12) | | | S(p=0.1516; f=0.11); NS(p=0.858; f=0.01) | | | ${OR}_{smokers}\approx{OR}_{never-smokers}$,  $p_{MH}$ = 0.967 | | | |  |
| *PSMB9* | *rs2127675* |  | n, % | **GG** | | **AG** | **AA** | **GG** | **AG** | **AA** | **AG/**  **AA** | **GG/**  **AA** | **AG/**  **AA** | **GG/**  **AA** | 0.946 |
|  |  | smokers | n | 56 | | 169 | 156 | 24 | 76 | 73 | **1.04**  (0.71; 1.53) | **1.08**  (0.64; 1.96) | **1.07**  (0.77; 1.49) | **1.03**  (0.64; 1.7) |  |
|  |  |  | % | *14.7* | | *44.4* | *40.9* | *13.9* | *43.9* | *42.2* |  |  |  |  |  |
|  |  | never-  smokers | n | 4 | | 27 | 26 | 15 | 67 | 74 | **1.15**  (0.61; 2.15) | **0.82**  (0.19; 2.31) |  |  |  |
|  |  |  | % | *7.0* | | *47.4* | *45.6* | *9.6* | *42.9* | *47.4* |  |  |  |  |  |
|  |  | HWE (p-val, f) | | | S(p=0.349; f=0.05); NS(p=0.385; f=-0.11) | | | S(p=0.5724; f=0.04); NS(p=0.966; f=0.00) | | | ${OR}_{smokers}\approx{OR}_{never-smokers}$,  $p_{MH}$ = 0.879 | | | |  |
| *PSMB9* | *rs17587* |  | n, % | **AA** | | **GA** | **GG** | **AA** | **GA** | **GG** | **GA/**  **GG** | **AA/**  **GG** | **GA/**  **GG** | **AA/**  **GG** | 0.765 |
|  |  | smokers | n | 37 | | 155 | 190 | 16 | 67 | 90 | **1.09**  (0.74; 1.61) | **1.08**  (0.59; 2.16) | **1.18**  (0.84; 1.63) | **1.05**  (0.61; 1.85) |  |
|  |  |  | % | *9.7* | | *40.6* | *49.7* | *9.2* | *38.7* | *52.0* |  |  |  |  |  |
|  |  | never-  smokers | n | 3 | | 26 | 28 | 11 | 57 | 88 | **1.43**  (0.76; 2.69) | **0.95**  (0.14; 2.92) |  |  |  |
|  |  |  | % | *5.3* | | *45.6* | *49.1* | *7.1* | *36.5* | *56.4* |  |  |  |  |  |
|  |  | HWE (p-val, f) | | | S(p=0.527; f=0.03); NS(p=0.331; f=-0.13) | | | S(p=0.489; f=0.05); NS(p=0.674; f=0.03) | | | ${OR}_{smokers}\approx{OR}_{never-smokers}$,  $p_{MH}$ = 0.759 | | | |  |
| *PSMB8* | *rs2071543* |  | n, % | **TT** | | **GT** | **GG** | **TT** | **GT** | **GG** | **GT/**  **GG** | **TT/**  **GG** | **GT/**  **GG** | **TT/**  **GG** | 0.560 |
|  |  | smokers | n | 5 | | 71 | 294 | 1 | 37 | 133 | **0.86**  (0.56; 1.37) | **1.66**  (0.42; 8.25) | **0.91**  (0.62; 1.33) | **1.78**  (0.53; 6.76) |  |
|  |  |  | % | *1.4* | | *19.2* | *79.5* | *0.6* | *21.6* | *77.8* |  |  |  |  |  |
|  |  | never-  smokers | n | 0 | | 14 | 43 | 0 | 38 | 117 | **1.02**  (2.29; 3.24) | **2.7**  (2.29; 3.24) |  |  |  |
|  |  |  | % | *0.0* | | *24.6* | *75.4* | *0.0* | *24.5* | *75.5* |  |  |  |  |  |
|  |  | HWE (p-val, f) | | | S(p=0.7588; f=0.02); NS(p=0.244; f=-0.14) | | | S(p=0.352; f=-0.07); NS(p=0.068; f=-0.14) | | | ${OR}_{smokers}\approx{OR}_{never-smokers}$,  $p_{MH}$ = 0.906 | | | |  |

Strength of association is measured with odds ratios (OR) for smokers (S) and never-smokers (NS) with 95% - confidence interval. OR.MH is common odds ratio estimated with the Mantel and Haenszel estimator which is useful when OR seems stable among smokers and never-smokers. This homogeneity of ORs was tested as H0: ${OR}_{smokers}={OR}_{never-smokers}$ vs. H1: ${OR}_{smokers}\neq{OR}_{never-smokers}$ and p-value is reported ($p_{MH}$). If true odds ratios among two strata are not identical but do not vary much, OR.MH still is a useful summary of the conditional associations between SNP and risk of cancer. Table also presents results of testing hypothesis H0: *There are no associations between genotype and risk of cancer, i.e. all* $ORs = 1$ opposite alternative H1: *H0 is false*, and reports them as *P**-values.

**Supplementary Table 6**. Expected age at diagnosis depending on genotype in SNPs related with risk of NSCLC. Analysis adjusted to sex and smoking status. F-test is ANOVA test for contrast constructed based on risks (ORs) and presented in Table 2. R^2^ is determination coefficient of the model. Data also presented on Fig. 2.

| **Gene** | **SNP** | **Smoking** | **Genotype** | **GG** | **GC** | **CC** | **F-test** | **R^2^** |
| --- | --- | --- | --- | --- | --- | --- | --- | --- |
| *ERAP 1* | *rs26653* | Smokers | Mean  CI95% | 64.39  63.26; 65.51 | 64.06  62.77; 65.35 | 61.62  58.27; 65.00 | F_1;456_ = 6.94  p = 0.0087 | 0.032 |
|  |  | Never-smokers | Mean  CI95% | 64.63  61.26; 68.00 | 63.22  58.40; 68.06 | 74.44  62.65; 86.21 |  |  |
|  | *rs26618* | Smoking | Genotype | CC | TC | TT | F_1;458_ = 1.93  p = 0.1654 | 0.022 |
|  |  | Smokers | Mean  CI95% | 64.02  60.50; 67.52 | 64.67  63.38; 65.97 | 63.66  62.56; 64.78 |  |  |
|  |  | Never-smokers | Mean  CI95% | 65.69  57.39; 73.97 | 63.07  58.51; 67.66 | 65.44  61.84; 69.04 |  |  |
|  | *rs2287987* | Smoking | Genotype | CC | TC | TT | F_1;435_ = 2.25  p = 0.1343 | 0.026 |
|  |  | Smokers | Mean  CI95% | 64.77  61.00; 68.57 | 64.16  62.69; 65.62 | 63.86  62.79; 64.93 |  |  |
|  |  | Never-smokers | Mean  CI95% | 62.28  52.13; 72.47 | 64.01  59.90; 68.08 | 65.44  61.68; 69.26 |  |  |
|  | *rs30187* | Smoking | Genotype | TT | CT | CC | F_1;495_ = 2.87  p = 0.0909 | 0.025 |
|  |  | Smokers | Mean  CI95% | 61.91  59.38; 64.49 | 64.09  62.86; 65.31 | 64.60  63.37; 65.83 |  |  |
|  |  | Never-smokers | Mean  CI95% | 66.12  57.01; 75.25 | 64.83  60.35; 69.30 | 64.33  60.73; 67.93 |  |  |
|  | *rs27044* | Smoking | Genotype | GG | CG | CC | F_1;456_ = 2.09  p = 0.1479 | 0.031 |
|  |  | Smokers | Mean  CI95% | 62.16  59.10; 65.23 | 63.86  62.54; 65.17 | 64.55  63.42; 65.67 |  |  |
|  |  | Never-smokers | Mean  CI95% | 67.84  53.37; 82.40 | 65.63  61.19; 70.06 | 63.82  60.39; 67.28 |  |  |

**Supplementary Table 7**. Linkage disequilibrium between SNPs in *ERAP1* and *ERAP2* genes among NSCLC patients and controls.

| SNP | LD | ***rs26653*** | ***rs26618*** | ***rs2287987*** | ***rs30187*** | ***rs27044*** | ***rs2248374*** |
| --- | --- | --- | --- | --- | --- | --- | --- |
| *ERAP1*  ***rs26653*** | D' | - | 0.923 | 0.999 | 0.556 | 0.172 | 0.362 |
|  | r |  | -0.319 | -0.317 | 0.474 | 0.171 | -0.208 |
| *ERAP1*  ***rs26618*** | D' | *0.999* | - | 0.999 | 0.977 | 0.999 | 0.649 |
|  | r | *-0.348* |  | -0.317 | -0.395 | -0.348 | 0.390 |
| *ERAP1*  ***rs2287987*** | D' | *0.954* | *0.956* | - | 0.999 | 0.960 | 0.049^*^ |
|  | r | *-0.316* | *-0.297* |  | -0.371 | -0.307 | 0.027^*^ |
| *ERAP1*  ***rs30187*** | D' | *0.632* | *0.999* | *0.999* | - | 0.973 | 0.468 |
|  | r | *0.531* | *-0.414* | *-0.393* |  | 0.837 | -0.315 |
| *ERAP1*  ***rs27044*** | D' | *0.285* | *0.999* | *0.925* | *0.977* | - | 0.632 |
|  | r | *0.281* | *-0.343* | *-0.302* | *0.810* |  | -0.366 |
| *ERAP2*  ***rs2248374*** | D' | *0.372* | *0.626* | *0.103^*^* | *0.324* | *0.512* | - |
|  | r | *-0.220* | *0.368* | *0.058^*^* | *-0.229* | *-0.300* |  |

* - not statistically significant at α=0.05

**Supplementary Figure 1.** Power of the chi-square test for association between genotype and risk of cancer. Assumptions: significance level $\alpha=0.05$, strata: smoking, $N_{Caces}=444$, $N_{Controls}=329$, additive model of the risk, i.e. $\ln\frac{\pi_{YY}}{1-\pi_{YY}}-\ln\frac{\pi_{XX}}{1-\pi_{XX}}=2\left[ \ln\frac{\pi_{XY}}{1-\pi_{XY}}-\ln\frac{\pi_{XX}}{1-\pi_{XX}} \right]$, where $\pi_{YY}, \pi_{XY}, \pi_{XX}$ is probability of cancer for individual with genotype $XX, XY, YY$, respectevitelly. Genotype $XX$ as baseline. Null hypothesis H0: *OR.XY/XX = OR.YY/XX = 1 both in smoking and never-smoking group*; alternative H1: *Risk increases with number of allels Y*. Power is probability (x100%) of rejection of a H0 depending on the strength of the association measured with ORs. Chi-square test on 2 degrees of freedom. Power vs. Odds Ratio YY/XX is presented. See also **Supplementary Table 4.**


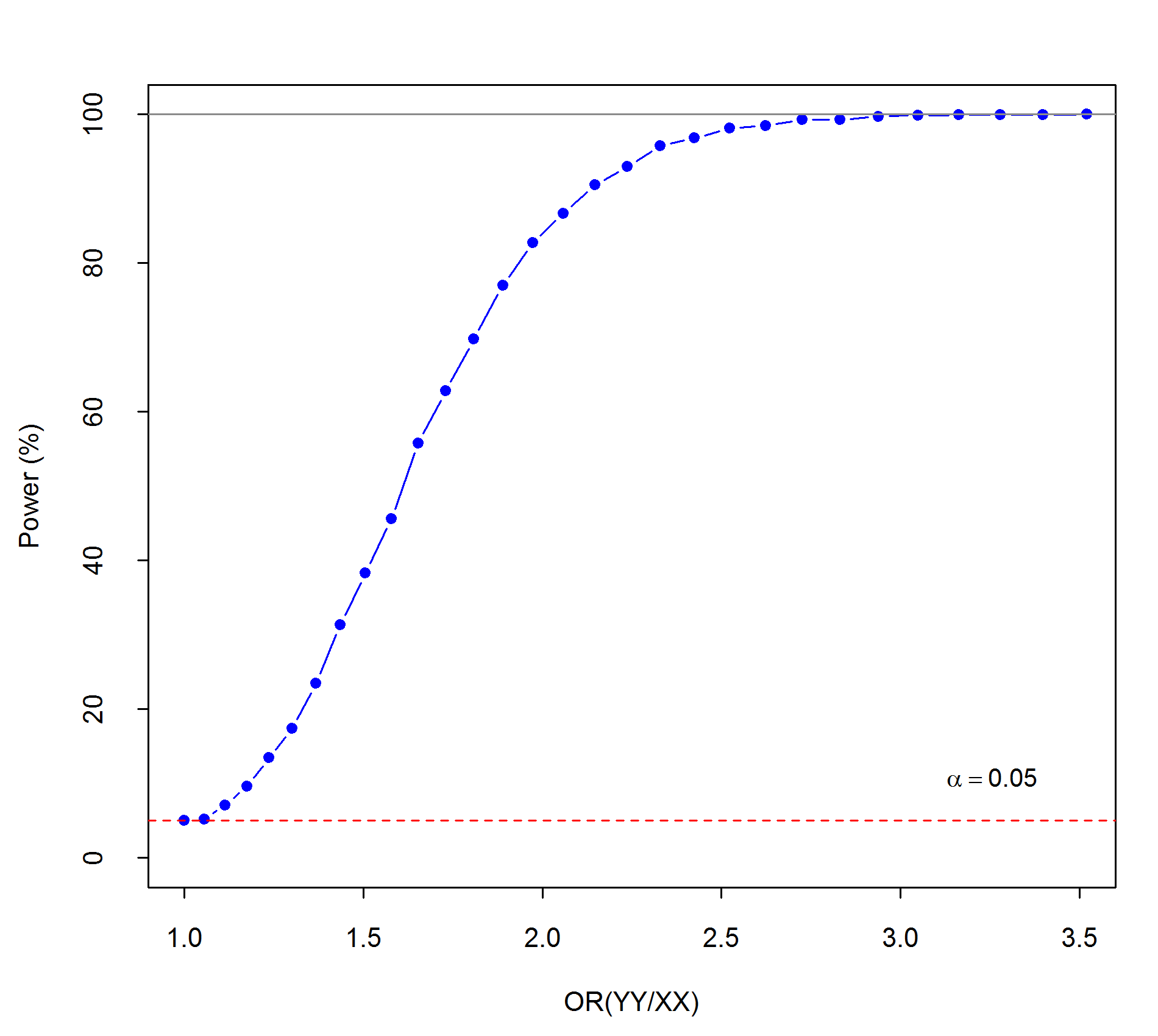

Supplement: Supplementary file 1 [file DataSheet_1.docx]
